# How climate change affects mosquito-borne infectious disease: The different trajectories of malaria and dengue

**DOI:** 10.1126/sciadv.aei0953

**Published:** 2026-10-02

**Authors:** Toby B Cumming, Henry Surendra, Karin Leder, Katherine L. Anders

**Affiliations:** ^1^ Health and Climate Initiative, Faculty of Medicine, Nursing and Health Sciences, Monash University, Melbourne, Australia.; ^2^ Division of Planetary Health, School of Public Health and Preventive Medicine, Monash University, Melbourne, Australia.; ^3^ Infectious and Tropical Diseases Epidemiology, Public Health Program, Monash University Indonesia, Tangerang, Indonesia.

## Abstract

As climate change increases temperatures, additional warmth combines with rainfall and humidity to enhance conditions for mosquito populations to thrive. Greater spread of *Anopheles* and *Aedes* vectors increases the risk of transmission of the world’s most prevalent mosquito-borne infectious diseases, malaria and dengue. Yet the impacts of climate drivers on disease transmission are complex: Relationships are nonlinear, effects are seasonal, and there is substantial local variability. Further complexity is added by the many nonclimate factors that influence disease transmission, including movement of people, population growth, immunity levels, land use change, urbanization, control interventions, and quality of health care systems. Here, we explain why, despite climate drivers having broadly similar effects on malaria and dengue transmission parameters, the trajectories of the two diseases differ, with rapid growth in dengue but long-term decline in malaria.

## INTRODUCTION

Climate change is increasingly claiming lives and harming people’s health (*1*). While it is accepted that climate change affects the spread of infectious diseases, determining exactly how is complicated, as there are more than 1000 unique pathways in which climate hazards alter disease transmission (*2*). In this narrative review, we focus on how climate change affects the world’s two most prevalent mosquito-borne infectious diseases, malaria and dengue. Malaria has an enormous burden of disease—282 million cases and 610,000 deaths annually (*3*)—that is concentrated in Africa. The malaria parasite (*Plasmodium*) is typically spread through the bite of infected female *Anopheles* mosquitoes. Once people are infected, they can carry the parasite in their blood for months or years. Malaria in Africa is mainly caused by *Plasmodium falciparum*, which is responsible for most severe and fatal cases. *Plasmodium vivax*, which is able to form dormant liver stages (called hypnozoites) that can reactivate long after initial infection to cause recurrent episodes, is more prominent in the Americas and Southeast Asia. In endemic areas, repeated exposure to the malaria parasite can lead to partial immunity against severe disease and death. Dengue, caused by a virus, occurs in tropical and subtropical regions of the Asia-Pacific, the Americas, and Africa and is transmitted to humans through the bite of infected female *Aedes* mosquitoes. The virus exists as four related serotypes and causes only transient infection, detectable in the blood for about a week. Many people infected with dengue virus do not exhibit symptoms, so quantifying the transmission rate is difficult. One modeling study estimated 390 million dengue virus infections globally per year, with 96 million having clinical symptoms (*4*). Immunity to dengue virus is predominantly serotype-specific, and secondary infection with a different serotype to the first is associated with an increased risk of severe disease (*5*).

The epidemiology of both malaria and dengue is shaped by the distribution of, and interactions between, the pathogens, their vector mosquitoes, and their hosts. Here, we consider the key climate drivers of transmission: temperature, precipitation, and humidity. Together, these drivers underpin climate impact pathways, including geographic limits, seasonality, mosquito abundance, and transmission intensity. We examine efforts to model global climatic suitability for malaria and dengue, used to infer current populations at risk and to project future transmission under climate change scenarios. Accurate global risk mapping is difficult, as substantial local variability in climate-driven risk interacts with a wide range of nonclimate effect modifiers (e.g., immunity levels, land use, population density, housing quality, drug and insecticide use and resistance, irrigation practices, and prevention programs). Ultimately, despite climate drivers having broadly similar effects on the transmission parameters for malaria and dengue, data indicate very different disease trajectories since the mid-20th century, with malaria in decline and dengue rapidly expanding (*6*). Nonclimate factors, including the long history of effective malaria control interventions and the differential effect of urbanization on malaria and dengue mosquito vectors, can explain much of the difference. In synthesizing the research evidence, we aim to show that, while malaria and dengue are undoubtedly climate-sensitive, the different biological features and ecological preferences of *Anopheles* and *Aedes* mosquitoes interact with both climate and nonclimate factors to drive differences in transmission risk.

## CLIMATE DRIVERS OF TRANSMISSION

Climate affects not only the mosquito vector, but also the pathogen and the host in ways that influence transmission risk (*7*). For mosquitoes, the traits most responsive to changes in climate include development rate, reproduction rate, mortality rate, and behavior (e.g., biting activity). For pathogens, climate-sensitive traits include replication rate and incubation period. For human hosts, traits include behavior (e.g., choice of clothing and sleeping habits) and level of immunity. Hosts can include nonhuman vertebrates, which can be reservoirs for zoonotic transmission [e.g., macaques in the case of malaria; (*8*)], and climate can influence transmission risk through changes in a host’s geographic distribution, population density, migration patterns, prevalence of infection, and pathogen load (*9*).

### Temperature

Of all the climate factors that affect mosquito-borne disease, temperature has the strongest evidence of association and is the most thoroughly investigated (albeit typically in controlled laboratory studies, without field validation). Mosquitoes are cold-blooded and small-bodied; their internal temperature is very sensitive to environmental temperature, and they typically fare better as temperatures rise. Temperature exerts an influence from the earliest stages of the mosquito lifecycle. *Anopheles gambiae*, the primary malaria vector in sub-Saharan Africa, develop from larvae into adults across a 16°–34°C temperature range; larval survival is >30 days at 14°–20°C, but <7 days at 10°–12°C and 38°–40°C (*10*). *Aedes aegypti*, the primary dengue vector in tropical urban environments, develop from larvae into adults in 7 days at 35°C but 40 days at 15°C, with 20°–30°C optimal for survival through all developmental stages (*11*). *Aedes albopictus*, a secondary vector for dengue, is better suited to temperate climates than *Ae. aegypti* (*12*). The biting rate of mosquitoes increases steadily with rising temperatures, for both *Anopheles* (*13*) and *Aedes* (*14*). Biting rate is related to the gonotrophic cycle (the time between a mosquito taking a blood meal and laying eggs), which accelerates as temperature increases (*15*). “Multiplicity of feeding” (mosquitoes taking blood meals from multiple hosts during a single gonotrophic cycle) has been linked to increasing temperature (*16*) and to higher levels of dengue transmission (*17*). For malaria, the extrinsic incubation period—beginning when the mosquito ingests infected blood and ending when the *Plasmodium* sporozoites (the first form of the malaria parasite) are fully developed and, thus, the mosquito is infectious—is shortest at 34°C (*13*). Dengue virus replication within the *Aedes* mosquito is also accelerated by higher temperatures: Average extrinsic incubation period is shorter at 30°C (6.5 days) than at 25°C (15 days) (*18*). Even a small reduction in this period can markedly boost pathogen transmission by increasing the number of mosquitoes that live long enough to become infectious.

While warmer temperatures are generally better for mosquitoes, there is a ceiling: Models combining key temperature-dependent parameters typically produce an inverted U-shape relationship between temperature and transmission risk (see Fig. 1). A 2013 study (*19*) showed that transmission potential of malaria is optimized at 25°–28°C, substantially lower than previous estimates. Another study corroborated this finding with a model indicating that 26°C was optimal for malaria transmission (*13*). Dengue modeling findings are similar, projecting maximal transmission at 26°–29°C (*20*) and maximal dengue outbreak risk between 27° and 31°C (*21*). One potential exacerbating factor not included in these models is human behavior: Rising temperatures make taking preventive actions such as wearing long clothes and sleeping under a mosquito net less appealing (*22*).

**Fig. 1.**
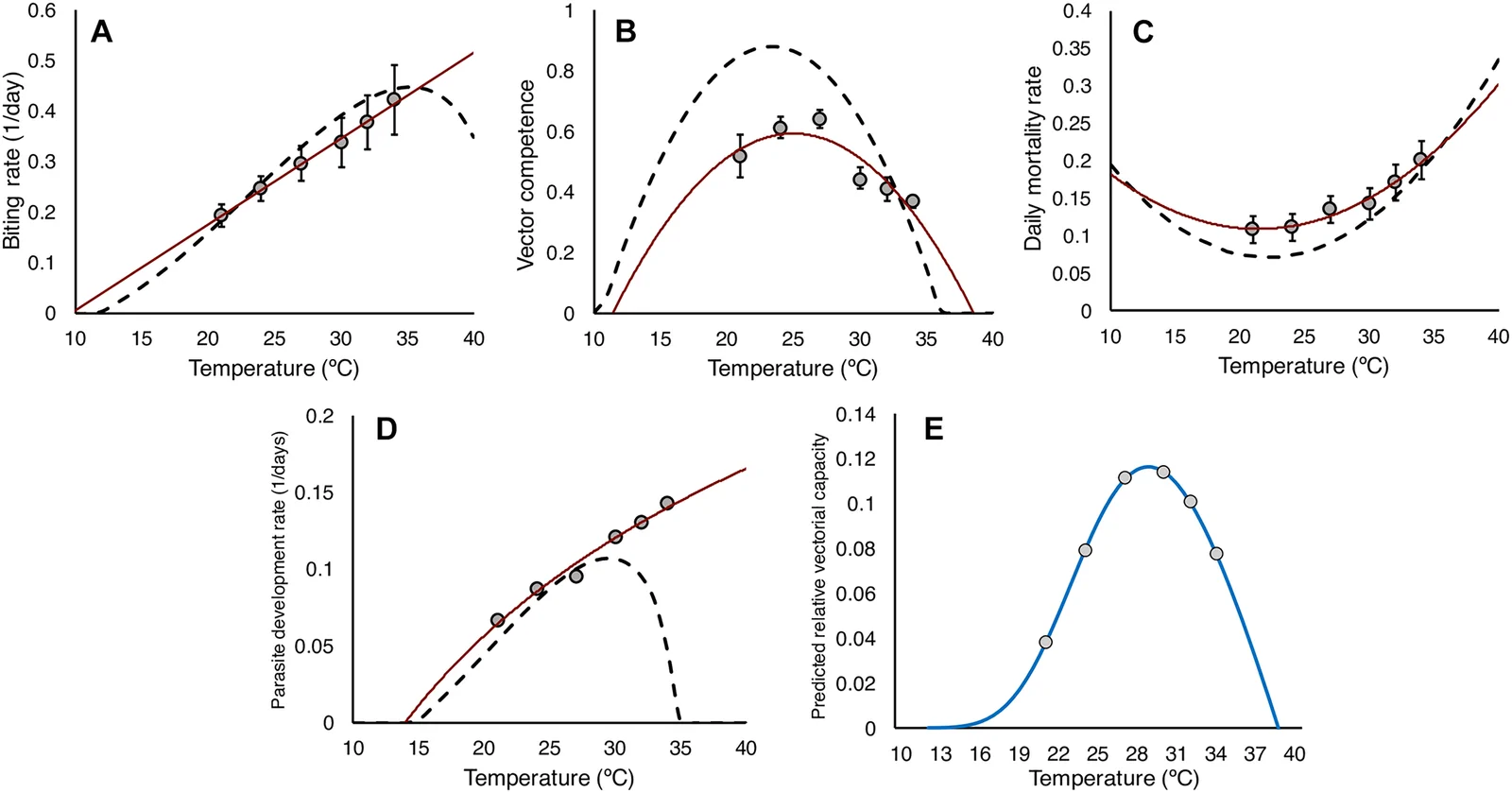
Thermal performance curves in the context of malaria. Including (**A**) biting rate, (**B**) vector competence, (**C**) mosquito mortality rate, (**D**) parasite development rate, and (**E**) the predicted relative vectorial capacity (rVC) based on these curves [data sources: red lines (*13*) and dashed black lines (*19*)]. Reproduced from Shapiro *et al.* (*13*) under CC BY 4.0 (https://creativecommons.org/licenses/by/4.0/deed.en).

Identifying “optimal” temperature for disease transmission is complicated by temperature fluctuations over time. At the same average temperature, *Ae. aegypti* mosquitoes exposed—under experimental conditions—to smaller daily temperature variability (“diurnal temperature range”) are more likely to become infected with dengue virus than those exposed to greater temperature variability (*23*). Reflective of tropical and temperate climates, dengue epidemic potential peaks at 29°C when diurnal temperature range is 0°C, but at 20°C when diurnal temperature range is 20°C (*24*). In a disease transmission model incorporating seasonal temperature variation, tropical and subtropical cities (e.g., Rio de Janeiro and Delhi) produce the largest epidemics, although more temperate cities (e.g., Shanghai) also have high epidemic suitability because large temperature variation offsets moderate average temperatures (*25*). Similar findings apply to malaria: With mean temperatures >21°C, diurnal temperature fluctuation slows parasite development compared with constant temperatures, whereas fluctuation speeds development at mean temperatures <21°C (*26*). While climate change is increasing average temperatures, it is decreasing diurnal temperature ranges due to night-time temperatures warming faster than day-time temperatures (*27*). A further consideration is ongoing mosquito adaptation: there is evidence for local climatic adaptation in *Ae. aegypti* populations (*28*), challenging the application of fixed thermal performance models to assess climate impact on mosquito-borne disease across space and time.

### Precipitation

Rainfall is strongly associated with transmission patterns of malaria and dengue, as it influences the availability, quality, and seasonality of sites for mosquitoes to lay eggs and for larvae to develop. Rainfall events spaced near the natural frequency of the mosquito reproductive cycle may produce “phase locking” and rapid population growth (*29*). *Anopheles* mosquitoes favor stagnant water for breeding, meaning that malaria incidence is closely tied to the rainy season and is higher in rural than urban areas (*30*). In contrast, the favored breeding sites for *Ae. aegypti* are artificial water-holding containers, which are plentiful in urban domestic environments. *Ae. aegypti* mosquitoes lay eggs on the inner walls of containers above the waterline, with larvae hatching once water rises to cover the eggs.

Malaria cases increase seasonally with times of high precipitation; this relationship appears to be stronger in Africa than in Asia (*31*). In West Africa, for example, cases peak in the rainy season from August to November (*32*). In Southern Africa, rainfall variability in December to February—associated with sea surface temperatures in the Eastern Pacific, a key parameter of the El Niño Southern Oscillation (ENSO)—accounts for more than two-thirds of the variability in malaria incidence from January to May (*33*). Interannual fluctuations in precipitation are also important: in Southern Africa, the years of highest malaria incidence are associated with above-average rainfall, while the lowest malaria years are associated with below-average rainfall (*34*). An increase in *Ae. aegypti* abundance and dengue incidence following the onset of the rainy season has been observed in Southeast Asia (*35*) and the Americas (*36*). Higher than average seasonal rainfall linked to El Niño events is associated with a higher *Ae. aegypti* infestation index in Brazil (*37*).

The association is not straightforward: precipitation data are often included in climate models as total or average rainfall over a certain time period, but both high and low rainfall can exacerbate malaria and dengue under certain conditions. Excessive rainfall can wash away eggs and larvae (*38*), and drought can paradoxically enhance breeding conditions by causing stagnant pools in water courses (malaria) and increased use of open water storage containers close to households (dengue). In Australia, the predicted expansion of *Ae. aegypti* range with climate change is driven by an assumed increase in household water storage in response to a drier climate (*39*). A Brazilian modeling study demonstrated that both wet conditions and extreme drought can increase dengue risk at different lags and that these relationships differ between rural and urbanized settings (see Fig. 2) (*40*). Similarly, in Barbados, dengue risk was associated with excess rainfall at lags up to 2 months, but also with drought at lags of 4 to 5 months (*41*). Data from the Philippines indicate that variability in dry season length can affect the relationship between rainfall and dengue incidence (*42*). For malaria, even in years where drought contributes to low incidence, this can suppress population immunity, thus making people more vulnerable once normal rainfall returns. In Venezuela, El Niño is associated with low rainfall, and the biggest increases in malaria incidence occur a year after recognized El Niño events (*43*). In epidemic years, malaria mortality is more strongly related to drought in the preceding year than to rainfall in the epidemic year. A further complication is that rainfall totals do not necessarily correspond with availability of surface water. Models have been created to relate precipitation to surface hydrology and, thus, mosquito breeding sites, down to the level of simulating individual ponds at the village scale (*44*). Bomblies and colleagues demonstrate that modeling the detailed hydrodynamics at village scale is necessary to explain both intervillage (*45*) and interseasonal (*46*) variation in mosquito abundance.

**Fig. 2.**
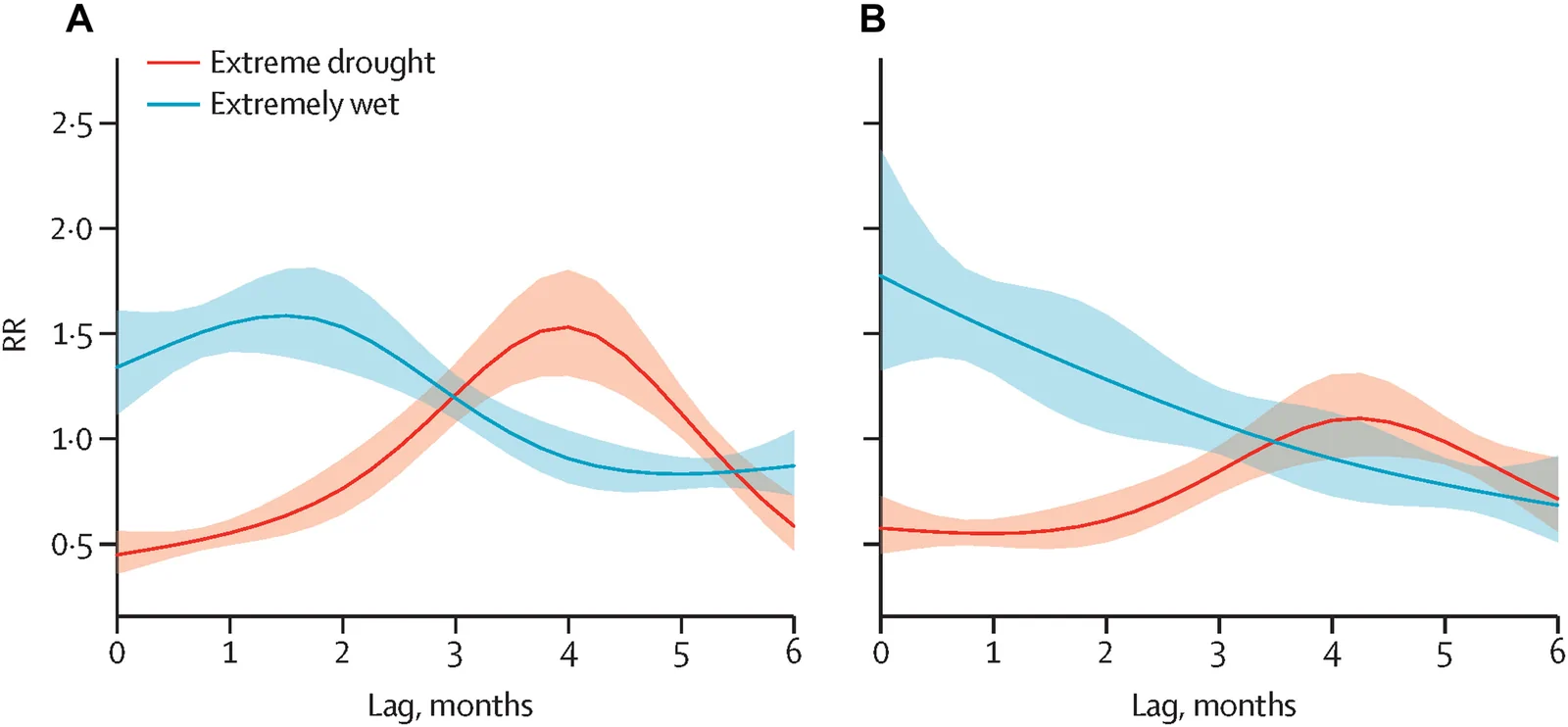
Dengue lag-response association for hydrometeorological hazard scenarios. For exceptionally wet (blue) and extreme drought (red) conditions at (**A**) high and (**B**) low levels of urbanization in Brazil. Shaded areas represent 95% CI. RR, relative risk. Reproduced from Lowe *et al.* (*40*) under CC BY 4.0 (https://creativecommons.org/licenses/by/4.0/deed.en).

### Humidity

Mosquitoes are very susceptible to desiccation—a loss of moisture that leads to dehydration—and thus need a certain level of humidity to thrive. Humidity is typically quantified as relative humidity (water content in the air as a percentage of the maximum it can hold at a specific temperature) to acknowledge its close inter-relationship with temperature. Higher relative humidity is linked to increases in mosquito activity (although not necessarily biting rate, which can be hydrating under dry conditions), egg production and longevity (*47*). A close association between relative humidity and malaria incidence has been established, with examples spanning India (*48*), Iran (*49*), and Zambia (*50*). Relative humidity levels of 55 to 80% are suitable for completion of the *Plasmodium* parasite life cycles (*48*). Humidity is also associated with dengue, above and beyond effects of temperature. In New Caledonia, for example, the best predictive variables for estimating dengue outbreaks (which typically peak in March to April) are maximal temperature in December and maximal relative humidity in October to December of the previous year (*51*). At the global scale, vapor pressure (a measure of humidity) can be used to model the geographical limits of dengue fever transmission (*52*). *Ae. aegypti* mortality risk rises with decreasing relative humidity (*53*). Not only can low relative humidity affect *Aedes* survival directly through desiccation of both eggs and mature adults, it can reduce it indirectly by increasing evaporation from containers in which larvae and pupae develop (*54*). *Ae. albopictus* mosquitoes are particularly vulnerable to low humidity: in the laboratory, their eggs show high mortality at ≤75% humidity, whereas in the field, numbers significantly decrease after dry periods (*55*).

### A historical note

The above sections have focused on optimal climate conditions for malaria and dengue, but disease transmission still occurs under climatically suboptimal conditions. Malaria was endemic and widespread in many temperate regions of the world until the mid-1900s, with major epidemics as far north as the Arctic Circle. The disease was common in the “marsh parishes” of England from the 16th to 18th centuries, despite this being a particularly cold period (*56*). Historical data from Finland indicate that semiactive, hibernating *Anopheles* mosquitoes survived the freezing winter in houses and stables (*57*). The same is true for humidity: *Anopheles* mosquitoes can survive in the severe drought and heat of Sudan’s arid zone, sheltering in dwelling huts and the cracks of dry wells (*58*). In temperate regions, *Ae. aegypti* eggs can survive the cold season by remaining viable in a quiescent state for several months, hatching when more favorable conditions return (*59*). A field study in Argentina found that low winter temperatures were not a barrier to survival for *Ae. aegypti* at the limit of its South American distribution (*60*). However, the egg banks may not tolerate all winters, and future scenarios suggest a limited shift in ecological niche beyond the tropics and subtropics (*61*). *Ae. albopictus* can better withstand low temperatures and overwintering—it is now established in many European countries, and future climate change may promote its establishment at higher latitudes in temperate regions (*62*).

### Extreme weather events

While climate change influences mosquito-borne disease risk through shifts in temperature, rainfall, and humidity, it also influences risk by increasing the likelihood of extreme weather events. Cyclone Idai in 2019 was linked to an almost threefold higher risk of malaria infection among affected households in Mozambique, with damage to housing appearing to drive the association (*63*). Extreme monsoon rainfall in 2022, heightened by climate change, brought Pakistan’s worst-ever flooding (*64*) and a subsequent fivefold increase in malaria cases (*65*). The majority of dengue cases in Peru’s 2023 epidemic were attributed to extreme precipitation during cyclone Yaku; climate forcing was shown to have increased the likelihood of concurrent extreme precipitation and warm temperatures in north-western Peru in March by 189% (*66*). Tropical cyclones in China’s Pearl River Delta between 2013 and 2018 were associated with an increased risk of dengue fever, with the largest relative risk of 1.62 [95% confidence interval (CI): 1.45–1.80] occurring at 5 days of lag (*67*). These extreme events interact with nonclimate factors, including population displacement, increased human-mosquito contact, and reduced access to healthcare, to exacerbate disease transmission.

## CLIMATE IMPACT PATHWAYS

Climate drivers converge to shape malaria and dengue transmission patterns by (i) dictating the geographic distribution of the relevant mosquito vectors and therefore the geographic limits of disease transmission, (ii) influencing mosquito life history and thus shaping mosquito abundance, seasonality, and duration of transmission season, and (iii) influencing vector competence through the influence of temperature on pathogen replication and dissemination in the mosquito. From 1951–1960 to 2015–2024, the climate-defined transmission potential of dengue increased by 12% for *Ae. aegypti* and 49% for *Ae. albopictus* (*1*). For malaria, there were small increases in climatically suitable areas for transmission over this time period: 2.0% for *P. falciparum* and 1.8% for *P. vivax* (*1*). The duration of the *P. falciparum* transmission season increased for 42% of malaria-affected areas, decreased for 30%, and remained stable for 28%; results for *P. vivax* were similar.

Dengue-affected areas are growing, with transmission expanding to higher altitudes and into more temperate regions. For example, dengue cases are increasing and expanding southward in Brazil (*68*) and the Asia-Pacific (*69*). Evidence from Brazil indicates that anomalously warm temperatures are driving the expansion (*70*). Dengue is also spreading in parts of the world not historically associated with the disease. This includes local transmission in areas without previous transmission [southern United States; (*71*)] and increased cases in areas with sporadic transmission [mainland France; (*72*)]. More months per year with suitable temperatures for *Aedes* mosquitoes mean a higher likelihood of dengue outbreaks, as illustrated by unprecedented climate-related outbreaks in Nepal (*73*).

For malaria, the evidence for climate-related geographic shifts in transmission is more contested. Historically, mountain communities—even those in equatorial regions—were too cold for *Anopheles* mosquitoes. Warming can change this, particularly increases in minimum temperature (*74*), but the data are inconsistent. There are clear associations in some contexts; for example, evidence from a 1987 Rwandan outbreak indicated that an increase in minimum temperature aligned with increased malaria incidence among people at high altitudes, who had little preexisting immunity (*75*). Similarly, a 2014 study showed that warming at high altitudes in Ethiopia and Colombia had exacerbated malaria outbreaks since the 1970s (*76*), with outbreaks appearing to move up and down the mountains in line with year-to-year temperature variations. Extrapolation of localized findings to all of Ethiopia’s highlands indicated that climate change had already contributed 5 to 6 million additional malaria cases (*77*). A model that included seasonality and climate variability explained 65 to 81% of the variance in monthly malaria outpatients across the East African highlands (*78*). Observed range limits for *Anopheles* mosquitoes in Africa were tracked from 1898 to 2016 using a compilation of datasets; findings suggested that the range shifted upward by 6.5-m elevation per year and southern limits moved poleward at 4.7 km per year (see Fig. 3) (*79*). However, it is difficult to isolate climate factors from behavior, ecology, and human immunity as drivers of malaria transmission dynamics (*80*). In the same East African highland region that yielded the above temperature-malaria association studies, others have found no link: In some areas, malaria increased without any change in climate variables (*81*), while in other areas, malaria decreased during periods of unambiguous warming (*82*).

**Fig. 3.**
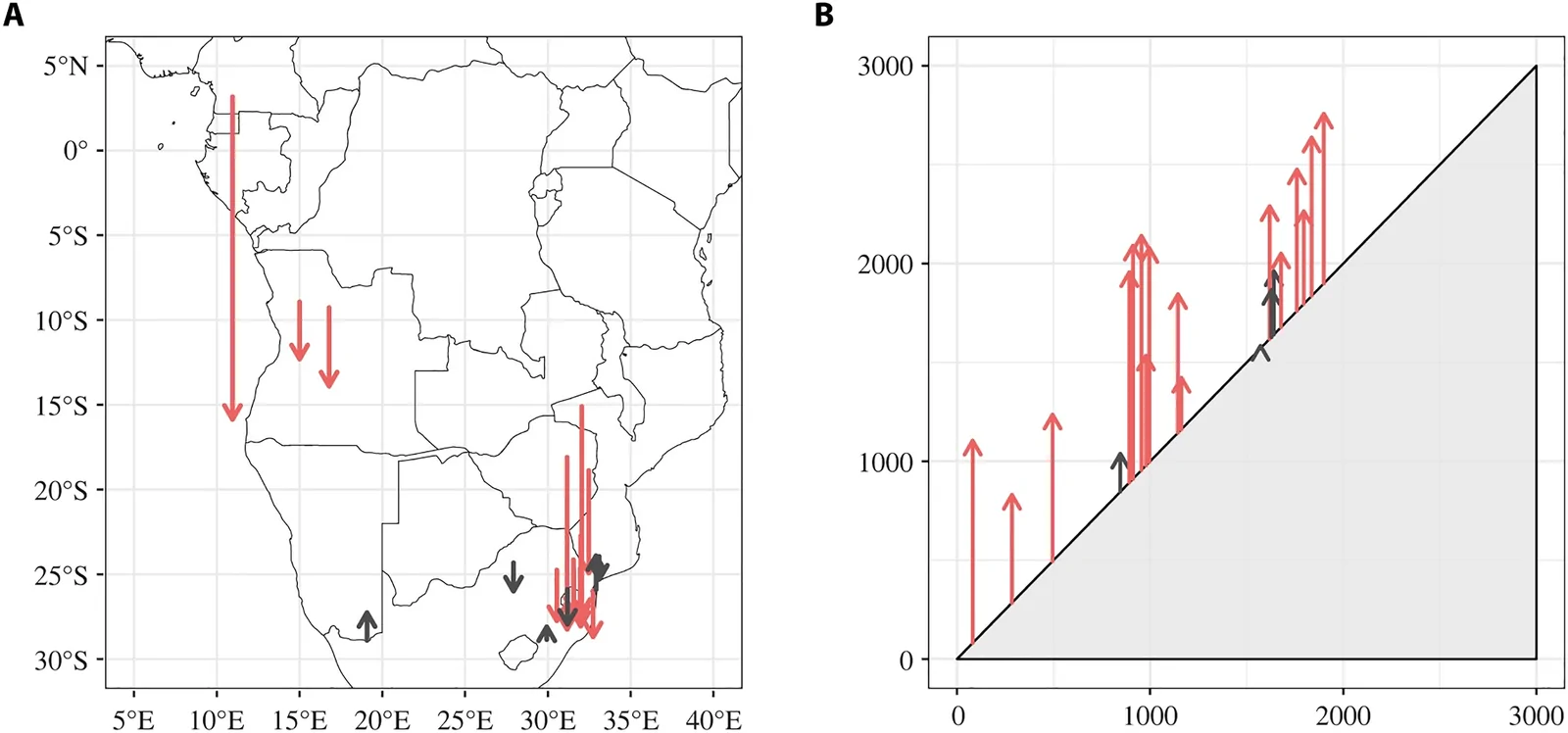
Estimated shifts in *Anopheles* species’ latitudinal and elevational maxima from 1900 to 2000. Each arrow gives one species’ estimate for (**A**) southern maxima and (**B**) elevation gain (in meters). Red arrows indicate statistically significant temporal trends (*P* < 0.05). Reproduced from Carlson *et al.* (*79*) under CC BY 4.0 (https://creativecommons.org/licenses/by/4.0/deed.en).

Some climate impact pathways take the form of periodic global cycles. ENSO is a climate cycle involving shifts in ocean temperature and atmospheric pressure in the tropical Pacific. ENSO has been linked to dengue, and to temperature and rainfall changes, in the South Pacific (*83*) and in South America (*84*). El Niño events are associated with increased transmission of dengue and malaria, particularly in Southeast Asia and Latin America (*85*). Detailed modeling of year-to-year variability in temperature and rainfall across highly divergent African regions, partly associated with ENSO, suggests that they are meaningful predictors of vector-borne disease (*86*). Temperature anomalies in the tropical Indian Ocean are also relevant; they have been used to predict worldwide dengue incidence from 1990 to 2019 (see Fig. 4) (*87*).

**Fig. 4.**
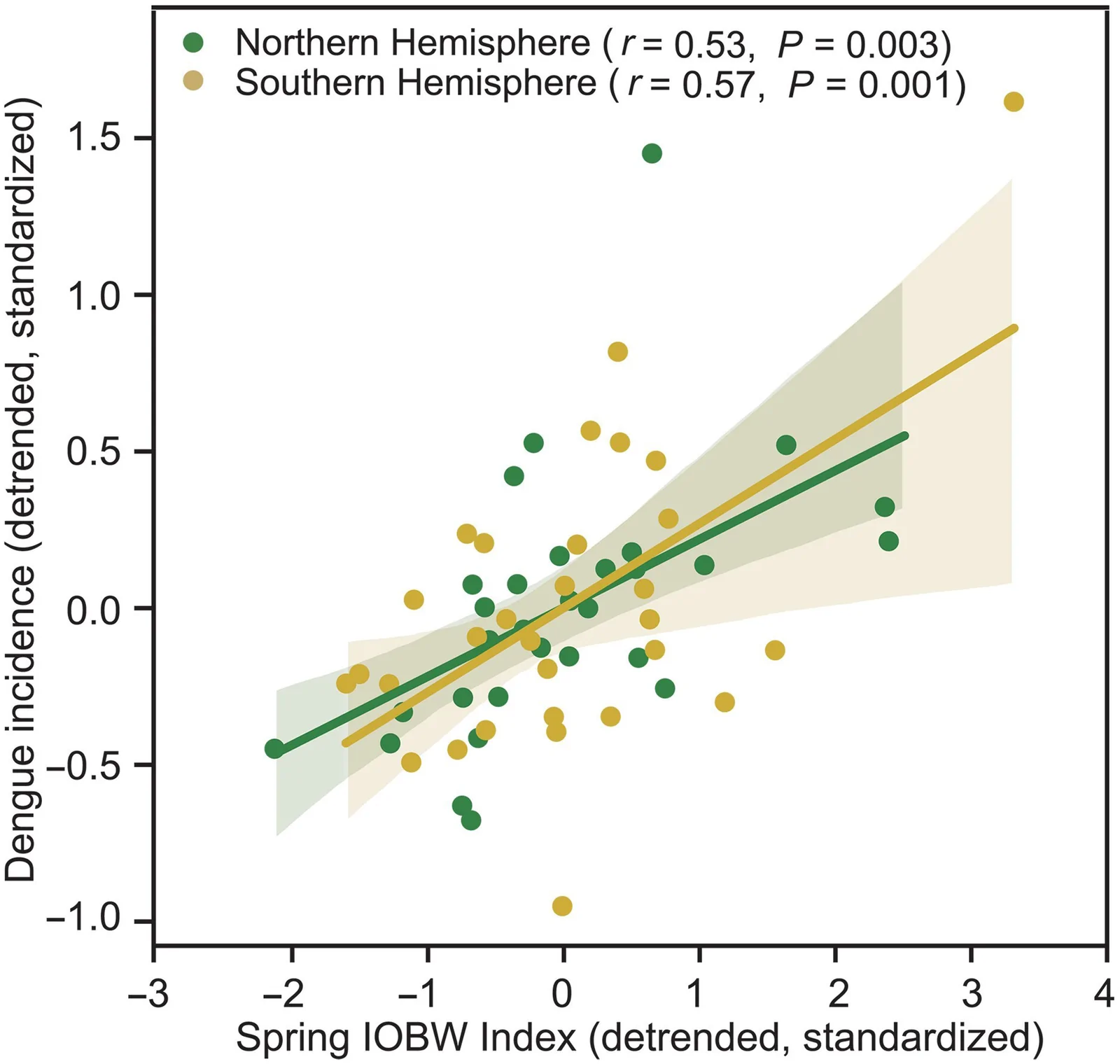
Association between the spring Indian Ocean basin-wide (IOBW) index and mean annual dengue incidence in the Northern and Southern Hemispheres, 1990–2019. Reproduced under license from Chen *et al.*, *SCIENCE*, doi:10.1126/science.adj4427, AAAS (2024) (*87*).

The influence of climate on transmission depends partly on the endemicity—or “stability”—of the disease. In areas of intense, stable malaria transmission, such as parts of sub-Saharan Africa where highly anthropophilic *Anopheles* mosquitoes deliver frequent infective bites, additional climatic suitability may have limited impact because transmission is already near saturation (*80*). Climatic variability therefore tends to have greater influence in low or unstable transmission settings, where malaria incidence fluctuates and epidemics occur. These epidemics, in settings with low population immunity, can result in more severe clinical outcomes and higher case fatality across a wider age range, compared with highly endemic areas where children are most affected. The interaction differs for dengue, where increasing transmission can amplify disease burden by increasing the frequency of secondary infection with a virus serotype different from the first, which is a risk factor for severe disease (*5*). Climate parameters have been used to successfully model the number, timing, and duration of dengue outbreaks (*88*), although outbreak cycles also depend on the dynamic immune profile of the population (*89*).

## GLOBAL RISK MAPPING AND FUTURE PROJECTIONS

Data on climate drivers and impact pathways have been used to project future mosquito-borne disease transmission. A prominent modeling study estimated that, by century end, malaria suitability will increase by 1.6 months per year in the tropical highlands of Africa, the Eastern Mediterranean, and the Americas, while dengue suitability will increase by 4.0 months per year in the lowlands of the Western Pacific and the Eastern Mediterranean (*90*). With the epidemic belt for both diseases expanding into temperate areas, an additional 4.7 billion people could be at risk of either malaria or dengue by 2070, relative to 1970–1999. This finding is consistent with earlier malaria modeling efforts, which projected both a geographical expansion and an increase in population at risk with climate change (*91*, *92*). Yet the first comprehensive intercomparison of models to evaluate the impact of climate change on malaria distribution was more equivocal. It predicted consistent increases in length of transmission season in highland regions, but consistent decreases in tropical regions, finding that the net effect of future climate change on population at risk was small (*93*). In Africa, projections indicate areas of expansion but also areas of contraction in previously endemic regions, including parts of the Sahel, due to rising temperatures and reductions in rainfall (*94*). Modeling work suggests that a 3°C increase in mean temperature from 27°C can reduce vectorial capacity (a summary measure of vector ability to transmit disease that integrates information on vector abundance, survival, competence, feeding rate, and extrinsic incubation period) by 51 to 89%, meaning that climate change might decrease malaria transmission in existing high transmission settings (*95*). Ryan and colleagues, who mapped transmission potential across Africa to 2080 under a midrange emissions scenario, predicted a small increase in total land area at risk for any malaria transmission, but a decrease in the net area suitable for intense, year-round transmission (*96*). Changes will be geographically specific. The population at risk of endemic malaria has been projected to decline out to 2080 in Central and Western Africa, but rise in Eastern Africa (*97*). In Asia, climate suitability for malaria is predicted to increase in Taiwan and parts of China, but decrease in India and much of Southeast Asia (*98*). The WHO and Lancet Commission have both concluded that, overall, long-term climate trends will assist rather than hinder malaria eradication efforts (*99*).

For dengue, there is widespread agreement that climate change will continue to drive increases in transmission. An early attempt to map the impact of climate change on future dengue transmission risk found that, at 2°C of warming, there would be expansion in disease extent, both in latitude and elevation, and a longer season of viral transmission in temperate regions (*100*). Climate-driven transmission risk was predicted to increase substantially, especially in Europe, North America, and high-altitude regions in the tropics, although this will be somewhat offset by reductions in climate suitability for *Ae. albopictus*, particularly in Southeast Asia and West Africa. Global abundance of *Ae. aegypti* rose steeply between 1990 and 2010, with further increases of 20 to 30% predicted out to 2080 (*101*). One study modeled the spread of *Ae. aegypti* and *Ae. albopictus* under different representative climate pathway (RCP) scenarios, with a particular emphasis on urbanization and human mobility, finding that distributions of both species will continue to expand, especially after 2050, into climatically suitable urban areas (*102*). Another study found that the increase in population at risk across the four different RCP scenarios was particularly strong for *Ae. aegypti* (see Fig. 5) (*103*). A modeling study that used RCP 6.0/shared socioeconomic pathway (SSP) 2 predicted that 2.3 billion more people will be at risk of dengue in 2080 compared to 2015, bringing the total population at risk to over 6.1 billion (*104*). Dengue suitability is influenced by temperature (68%), precipitation (13%), humidity (6%), and gross domestic product (GDP) (5%), and expansion will be driven more by population growth in already endemic areas than by spread to new populations. At a regional level, climate models have predicted increasing risk of dengue transmission in Hawaii (*105*), China (*106*), and Korea (*107*). Ecuador, where geographic range for dengue is projected to reduce with future climate change, is a rare exception (*108*). The impact of emissions mitigation on dengue incidence across Latin America has been quantified: Limiting global warming to 2°C could mean 2.8 million fewer dengue cases per year by 2100, compared with a no-policy scenario of +3.7°C (*109*).

**Fig. 5.**
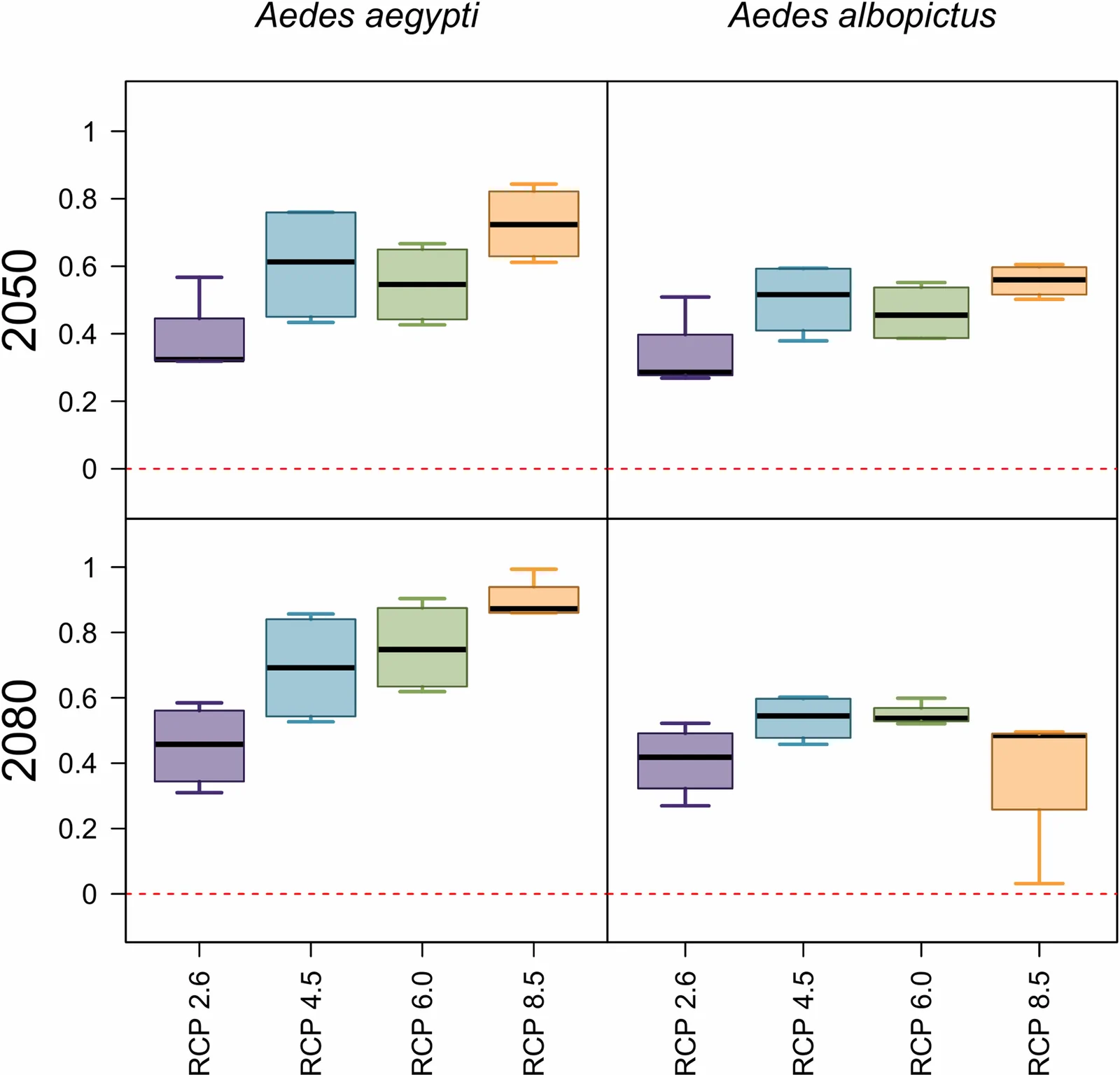
Projected changes in population at risk of *Aedes*-borne virus exposure. Net difference in billions at risk between current levels and 2050 (top row) and 2080 (bottom row) for *Ae. aegypti* and *Ae. albopictus* transmission at different RCPs. Reproduced from Ryan *et al.* (*103*) under CC BY 4.0 (https://creativecommons.org/licenses/by/4.0/deed.en).

The question of whether climate change will expand malaria and dengue transmission into more temperate areas has often focused on Europe. Some have predicted growing suitability for malaria in Europe, with a northward spread of *Anopheles* mosquitoes and extension of season that enables transmission for up to 6 months a year during 2051–2080 (*110*). Others argue that models driven principally by temperature are insufficient, that more realistic multivariate models suggest that expansion into Europe is unlikely (*111*), and that effective health surveillance systems are likely to mitigate the risk of any increase in climate suitability translating into increased disease burden (*93*). Much will depend not just on temperature, but on “receptivity” (are the vector, human host, and ecological conditions conducive to sustaining onward transmission following pathogen introduction?) and vulnerability to importation risk at the fringes of current transmission (*110*). For dengue, analysis based on historic and projected temperatures (1901–2099) shows a seasonal spike in vectorial capacity in summer across 10 European cities and predicts future increases in transmission intensity and epidemic potential, especially under a more extreme climate scenario (RCP 8.5) (*112*). Temperature-based modeling suggests a growing threat of dengue in Europe, with parts of the Mediterranean particularly at risk (*113*). *Ae. albopictus* (which plays a minor part in epidemics elsewhere in the world but is the main established vector in Europe) has potential to further expand its range in Europe because of its overwintering capabilities and its ecological plasticity in the context of climate change (*114*). Increasingly favorable climatic conditions in Europe are driving the trend for shorter intervals between regional establishment of *Ae. albopictus* and outbreaks of dengue (*115*). A modeling study that included both climatic variables and socioeconomic factors to estimate disease occurrence (rather than mosquito abundance) under climate change scenarios projected dengue hot spots in Southern Europe, especially in coastal areas (*116*).

## NONCLIMATE MODIFIERS OF RISK

There are challenges to modeling climate impacts at the global scale; local weather patterns are strongly influenced by subtle changes in vegetation, topography, water bodies, and coastlines (*117*). To predict vectorial capacity and transmission accurately at local levels requires downscaling to achieve sufficient spatial resolution, enabling consideration of microclimatic influences on key mosquito and pathogen traits. However, perhaps the greatest complication in determining climate impacts on malaria and dengue, beyond data limitations and methodological challenges, is accounting for the interdependent effects of the many nonclimate variables that influence transmission. Nonclimate factors can be epidemiological, ecological, or socioeconomic (*118*). Overlaying such factors on a temperature-dependent framework is critical for understanding the likelihood and magnitude of outbreaks (*25*). Ultimately, “a suitable climate is necessary but often not sufficient, as the climate determines the potential geographic distribution of a vector-borne disease, but other factors determine the actual distribution within the boundaries set by climate” (*119*).

In 2008, at a time when some were predicting great expansion of malaria with climate change, Reiter (*80*) warned against neglecting the key factors in transmission and epidemiology of malaria, including the ecology and behavior of both humans and vectors, and the immunity of the human population. These key nonclimate factors included birth rate, forest clearance, agriculture, movement of people, urbanization, insecticide resistance, drug resistance, degradation of health infrastructure, war, and civil strife. Influential nonclimate drivers have been classified into: vector and pathogen characteristics (insecticide and pathogen drug resistance, vector, and pathogen evolution), globalization and environment factors (ecosystem degradation, international travel and trade, urbanization, and population displacement), sociodemographic variables (population demographics, level of economic development, baseline disease incidence, population health status, and humanitarian crises), and public health system features (surveillance, early warning systems, vector control, quality of healthcare system, and research) (*119*). These factors interact with each other, and with climate drivers, to influence transmission of mosquito-borne disease in highly complex ways, sometimes favoring expansion and sometimes favoring contraction. One example of a model that addresses this complexity is HYDREMATS, which incorporates key hydrological, entomological, and immunological processes—rather than simpler climate inputs like average temperature and rainfall—linking environmental variables to malaria infections in humans. Outputs from this model suggest no major increases in the frequency or severity of malaria outbreaks with climate change in West Africa out to 2100 (*120*).

One fundamental macroscopic factor is globalization and the increased human mobility that has enabled the spread of both mosquitoes and hosts. International travel by ship aided the global dispersal of *Ae. albopictus*, while *An. gambiae*—the primary vector of malaria in Africa—was introduced to Brazil in the 1930s and thrived in a climate similar to its native Kenya (*121*). Mobility also drives transmission on smaller scales; for example, human movement predicts the geographic spread and timing of dengue epidemics in Pakistan (*122*). Another important nonclimate factor is human behavior, which plays a critical role in determining host-vector contact and thus vector-borne disease transmission (*123*). Human activities, water storage practices, household surroundings, and proximity to livestock or crops can all increase the risk of exposure to mosquito-borne disease (*124*). A central underlying driver is socioeconomic development. Infectious diseases of poverty, a classification that includes malaria and dengue, disproportionately affect the world’s poorest populations, people who typically have inadequate access to health services, poor housing, and are living under weak governance structures (*125*). Brazilian data indicate that socioeconomic variables are just as important as environmental variables in estimating infectious disease risk, particularly for dengue, with an inverse relationship between poverty and disease transmission (*126*). Global distribution of malaria can be modeled using temperature, precipitation, and a single nonclimate variable: per capita GDP (*127*). Climate factors had a greater impact on malaria transmission in countries with low per capita GDP (<US $20,000), suggesting that socioeconomic development can counteract climate-driven effects. Projecting out to 2050, the population at risk of malaria was 5.2 billion in a model with only climate factors, but just 2.0 billion when the model combined climate factors and GDP (*127*). GDP is likely to be a proxy for many aspects of welfare, economic status, and preventive programs relevant to malaria risk. A comprehensive approach to malaria prediction in Africa incorporated socioeconomic indicators and levels of intervention coverage alongside climate indicators and development pathways (*128*). Under a midrange climate scenario (SSP 2), and assuming current levels of intervention coverage, this analysis predicted a decrease in malaria incidence out to 2050 (although total cases would stay relatively stable due to population growth).

## MALARIA IN DECLINE, DENGUE ON THE RISE

Climate factors tend to push the biological processes underlying malaria and dengue in the same direction: Warmer temperatures, sufficient rainfall, and high humidity foster mosquito populations and boost disease transmission. However, rates of malaria have been falling over the last century, while rates of dengue have been rising. Global analysis reveals a decreasing trend in malaria, in both its endemicity and geographic extent, since 1900 (*129*). Endemic malaria is likely to have covered 58% of the world’s land surface around 1900 but only 30% by 2007, yet even more marked is the drop in prevalence within this reduced range (see Fig. 6). Gething and colleagues concluded that “predictions of an intensification of malaria in a warmer world, based on extrapolated empirical relationships or biological mechanisms, must be set against a context of a century of warming that has seen marked global declines in the disease and a substantial weakening of the global correlation between malaria endemicity and climate” (*129*). Contrary to suggestions that the margins of current disease extent will be most sensitive to the effects of warming [for example, (*91*)], observations indicate that the largest declines in malaria transmission are in these marginal zones, concurrent with increased intervention activity [for example, (*130*)]. *P. falciparum* infection prevalence in Africa declined from 40% in 1900–1929 to 24% in 2010–2015, with trends and cycles in malaria transmission over this time not explained by climate or intervention alone (*131*). The reduction in malaria from 2000 to 2015 was unprecedented, with new cases per 1000 people at risk declining from 79 to 59, although the subsequent increase to 64 in 2024 indicates that continued progress cannot be assumed (in 18 countries, incidence increased by ≥70% between 2015 and 2024) (*3*). A recent spatial and temporal mapping analysis noted a similar pattern, with a plateau in case incidence rates of malaria since 2015 (*132*).

**Fig. 6.**
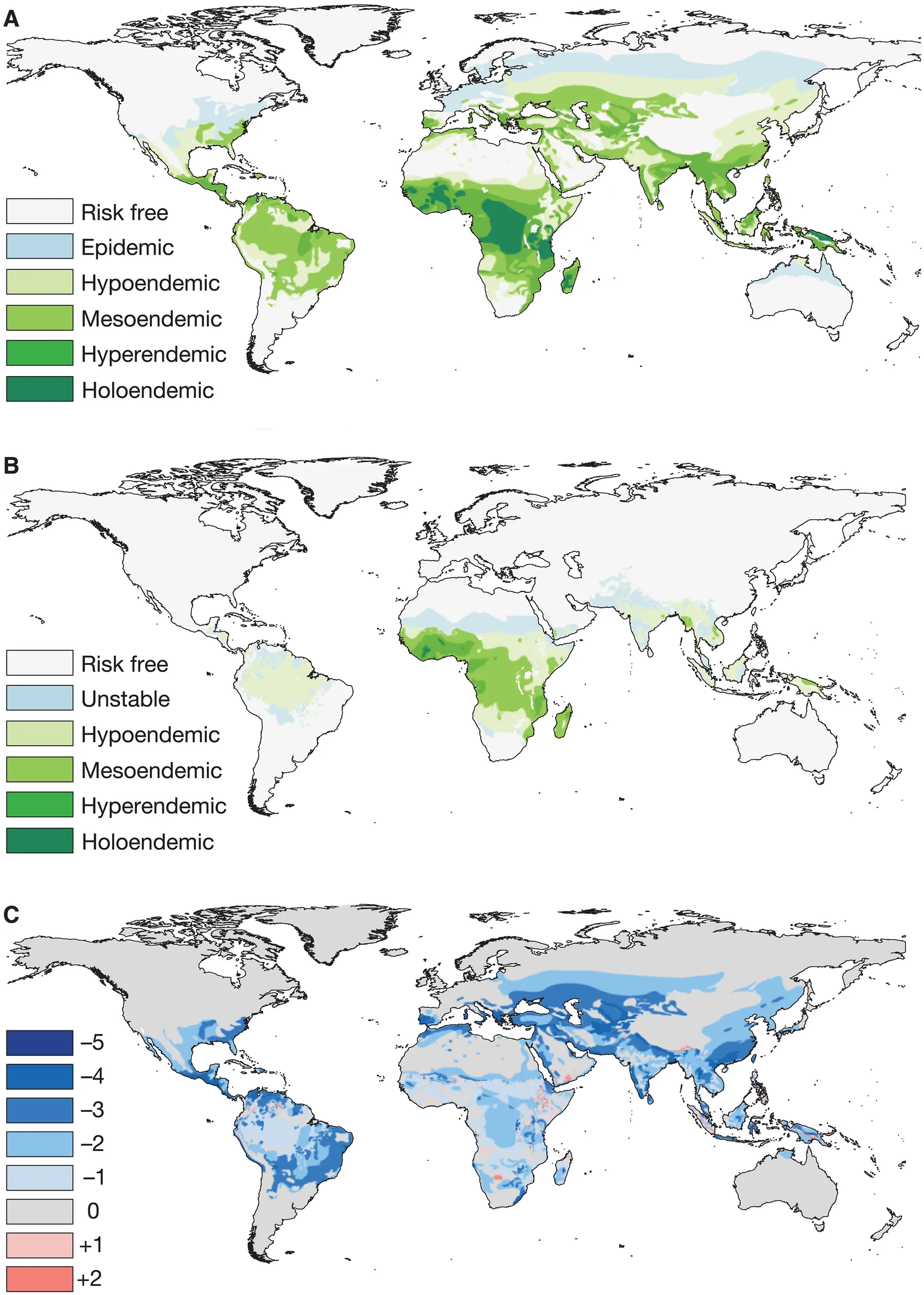
Changing global malaria endemicity from 1900 to 2007. (**A**) Preintervention endemicity at ∼1900, (**B**) contemporary endemicity at 2007, and (**C**) change in endemicity class between 1900 and 2007 (negative values denote a reduction, positive values an increase). Reproduced under license from Gething *et al.*, *NATURE*, doi:10.1038/nature09098, Springer Nature (2010) (*129*).

In contrast to malaria, dengue is expanding. More than 14 million dengue cases were reported to WHO worldwide in 2024, more than doubling the previous annual record (*133*). Recent years have seen increases in dengue across many equatorial regions, including the Americas, Southeast Asia, and the Pacific. Worldwide, the annual estimated dengue burden increased from 23.3 million cases in 1990 to 104.8 million in 2017, with age-standardized incidence rate increasing from 432 to 1371 per 100,000 population during this period (*134*). The increase in dengue coincides with the steady increase in climate suitability for *Ae. aegypti* and *Ae. albopictus* since the 1980s (*1*). While acknowledging the multifaceted causes, including population growth, increased human mobility, rapid urbanization, and improved diagnostic capacity, a 2019 review concluded that climate change is driving, and will continue to drive, rising dengue incidence (*135*).

There is the possibility that sub-Saharan Africa might see a shift in disease burden from malaria to dengue (and other arboviruses), due to climate-driven changes in environmental suitability for the respective mosquito species (*136*). Nonclimate factors, however, are likely to explain much of the divergent trajectories of the two diseases (see Table 1). The decline in malaria transmission in Europe beginning in the 19th century was not driven by temperatures (which were warming), but by improved socioeconomic conditions, better health care, and enhanced irrigation and drainage (*56*). The availability of effective vector control and pharmaceutical interventions is one particularly important difference between malaria and dengue. Concerted malaria eradication attempts extend back to the 1950s, with indoor residual spraying and mass drug administration programs using chloroquine and pyrimethamine, and, more recently, artemisinin combination therapy. Antimalarial drugs, for both prophylaxis and treatment, and widespread implementation of preventive strategies such as insecticide-treated bed nets and household spraying of residual insecticides have reduced the burden of malaria (*137*). Between 2000 and 2016, global spending on malaria increased from US $1.5 billion to $4.3 billion, underpinning a 36% decline in annual incidence rate and a 60% decline in annual death rate (*138*). For dengue, despite substantial increases in therapeutics research and development funding in recent years, its global expansion continues amid a lack of effective antiviral drugs and vaccines, which are only now beginning to emerge (*139*). While bed nets have proved an effective strategy for malaria control and the night-biting *Anopheles* mosquitoes, the day-biting behavior of *Aedes* mosquitoes makes this an ineffective tool for dengue.

**Table 1.** Common, malaria-specific, and dengue-specific effects of climate and nonclimate factors.

	Common effects	Malaria-specific	Dengue-specific
Mosquito vector		*Anopheles*	*Aedes*
Pathogen		*Plasmodium* parasite	Dengue virus
Climate factors			
Temperature	Warmer temperatures (up to an optimum) = faster larval development and greater survival (*10*, *11*), higher biting rate (*13*, *14*), and accelerated extrinsic incubation period (*18*).*	Transmission optimized at 25°–28°C (*19*), extrinsic incubation period shortest at 34°C (*13*).	Transmission optimized at 26°–29°C (*20*), maximal outbreak risk at 27°–31°C (*21*). *Ae. albopictus* better suited to cooler climates than *Ae. aegypti* (*12*).
Precipitation	Higher rainfall = greater availability of sites for breeding and larval development (*29*).†	Paradoxically, drought can enhance conditions by creating stagnant pools and can suppress population immunity, leaving people more vulnerable once rainfall returns (*43*).	Paradoxically, drought can increase mosquito breeding through increased use of water storage containers close to households (*39*).
Humidity	Higher humidity = greater mosquito activity, egg production, and adult survival (*47*).	Relative humidity of 55 to 80% is suitable for the *Plasmodium* parasite (*48*).	Low humidity can increase evaporation from containers, reducing larval development sites (*54*). *Ae. albopictus* particularly vulnerable to low humidity (*55*).
Nonclimate factors			
Population growth	Population growth = greater number of potential hosts. Increasing population at risk in existing endemic areas.	Population growth in sub-Saharan Africa and stalled decline in malaria incidence since 2015 = increasing global case numbers (*132*).	Projected increases in people at risk of dengue by 2080 mostly driven by population growth in already endemic areas (*104*).
Human mobility	Greater host mobility = higher risk of transmission (*122*).		
Human behavior	Choice of clothing and proximity to livestock and crops affect risk of vector-host contact (*123*).	Sleeping habits affect risk of vector-host contact (*Anopheles* are night-biting) (*123*).	Water storage habits affect risk of vector-host contact (*124*).
Immunity		Partial immunity after repeated malaria exposure can protect against severe disease, although there are high-risk groups (e.g., children <5 and pregnant women) (*137*).	Prior dengue virus exposure is associated with a greater risk of severe disease with second infection from different virus serotype (*5*).
Urbanization		Greater urbanization = fewer breeding sites suitable for *Anopheles* mosquitoes (*144*) [although *An. stephensi* is a highly competent urban malaria vector (*147*)].	Greater urbanization = more breeding sites suitable for *Aedes* mosquitoes (*140*).
Established vector control interventions		Successful use of insecticide-treated bed nets and household spraying (*137*).	Mosquito modification approaches [e.g., with *Wolbachia* (*153*)] are emerging.
Pharmaceutical interventions		Effective use of antimalarial drugs for prophylaxis and treatment (*137*), although drug resistance a concern. Malaria vaccine being rolled out in parts of Africa.	Dengue vaccines licensed in multiple countries but not yet in wide programmatic use (*151*). No licensed therapeutic drugs.
Health system capacity	Greater capacity = better diagnosis, treatment, and surveillance.	Clear treatment options (*137*).	Historic diagnostic uncertainty, limited treatment options (*139*).

* NB: Associations are nonlinear. Diurnal temperature variability is also important.

† NB: Excessive rainfall can wash away eggs and larvae. Context-dependent associations, with complex lag effects.

A second key disparity between malaria and dengue is the differential impact of urbanization on their respective vector mosquito species. Urban expansion has promoted the spread of dengue (*140*), with both *Ae. aegypti* and *Ae. albopictus* well adapted to cities, where there are numerous egg-laying sites (such as gutters and water containers), little risk of predation, and plentiful human blood meals. Data from China indicate that *Ae. albopictus* have higher density, faster larval development rate, and longer adult survival time in urban areas than in suburban and rural areas (*141*). In Singapore, *Ae. aegypti* abundance is related to features of the urban environment, including the presence of gully traps for drainage (*142*). A Taiwanese study found that urbanization was the strongest explanatory factor for dengue among the social and demographic variables tested (*143*). Global warming is interacting with increased urbanization and international travel to boost dengue transmission risk (*128*). In contrast, urbanization appears to reduce malaria transmission (*144*), both for *P. falciparum* (*145*) and *P. vivax* (*146*), most likely because cities lack the more natural breeding sites and larval habitats favored by *Anopheles* mosquitoes. There is one important caveat: *Anopheles stephensi*, native to parts of Asia and the Middle East, is a highly competent urban malaria vector that is invading Africa and is now firmly established in urban settings in the Horn of Africa (*147*). *An. stephensi* thrives in artificial water containers, making it well adapted to cities and less restricted by dry seasons, and also shows resistance to common insecticides (*148*).

## CONCLUSION

In many regions of the world, climate change–induced warming creates conditions that favor transmission of mosquito-borne diseases. Many physiological traits of malaria and dengue—most importantly, how long the mosquito vectors are alive, infectious, and biting—are driven by temperature, as is the speed that the pathogen can develop and be transmitted from the mosquito to a human host and back again. Projections suggest that climate change will continue to expand the geographic range that is “suitable for transmission.” Yet ultimately what drives the incidence of malaria and dengue is a complex mix of climate variables, localized environmental conditions, and a multitude of nonclimate factors, including land use change, urbanization, population growth, movement of people, immunity of the population, insecticide and drug resistance, control interventions, and quality of health systems. Mapping and understanding the trajectories of malaria and dengue has implications for prioritizing vector control and disease prevention initiatives. Although malaria is in long-term decline, data indicate a rise in incidence of 8.5% over the past decade (*3*). Most resurgence in malaria over the last century has been associated with a weakening of malaria control measures, often due to resource constraints, but also due to war or disaster (*149*). Beyond the ecological mechanisms of climate change, disruptions caused by extreme weather events may emerge as a primary driver of malaria risk in Africa (*150*). We must maintain and expand the malaria intervention programs that have established efficacy, reducing the burden even in the face of global warming. For dengue, the rapidly rising burden is a serious concern, and we must escalate our prevention and control efforts. Novel interventions are emerging for dengue that include partially effective vaccines and innovative mosquito control strategies (*151*). A live, attenuated, tetravalent dengue vaccine (Qdenga) has been licensed in many countries since 2022, while another (Butantan) has recently shown a strong efficacy and safety profile (*152*). Among mosquito modification approaches, one particularly promising strategy is introducing the bacterium *Wolbachia* into *Ae. aegypti* populations to block transmission of dengue (*153*). Ideally, mosquito-borne disease prevention should combine multiple synergistic interventions to reduce reliance on chemically based approaches and counter the problem of insecticide and drug resistance that occurs with stand-alone interventions (*154*). By accelerating our efforts in prevention, vector control, and vaccine development while also cutting our greenhouse gas emissions to limit global warming, we can minimize the future burden of malaria and dengue.

## References

[1] M. Romanello, M. Walawender, S.-C. Hsu, A. Moskeland, Y. Palmeiro-Silva, D. Scamman, J. W. Smallcombe, S. Abdullah, M. Ades, A. Al-Maruf, N. Ameli, D. Angelova, S. Ayeb-Karlsson, J. Ballester, X. Basagaña, H. Bechara, P. J. Beggs, W. Cai, D. Campbell-Lendrum, G. E. C. Charnley, O. Courtenay, T. J. Cross, C. Dalin, N. Dasandi, S. Dasgupta, M. Davies, M. Eckelman, C. Freyberg, P. G. Corral, O. Gasparyan, J. Giguere, G. Gordon-Strachan, S. Gumy, S. H. Gunther, I. Hamilton, Y. Hang, R. Hänninen, S. Hartinger, K. He, J. Heidecke, J. J. Hess, S. Jankin, O. Jay, D. K. Pantera, I. Kelman, H. Kennard, G. Kiesewetter, P. Kinney, D. Kniveton, V. Koubi, R. Kouznetsov, P. Lampard, J. K. W. Lee, B. Lemke, B. Li, A. Linke, Y. Liu, Z. Liu, R. Lowe, S. Ma, T. Mabhaudhi, C. Maia, A. Markandya, G. Martin, J. Martinez-Urtaza, M. Maslin, L. McAllister, C. McMichael, Z. Mi, J. Milner, K. Minor, J. Minx, N. Mohajeri, N. C. Momen, M. Moradi-Lakeh, K. Morrisey, S. Munzert, K. A. Murray, N. Obradovich, P. Orgen, M. Otto, F. Owfi, O. L. Pearman, F. Pega, A. J. Pershing, A.-C. Pinho-Gomes, J. Ponmattam, M. Rabbaniha, T. Repke, J. Roa, E. Robinson, J. Rocklöv, D. Rojas-Rueda, J. Ruiz-Cabrejos, M. Rusticucci, R. N. Salas, A. S. J. Plana, J. C. Semenza, J. D. Sherman, J. Shumake-Guillemot, P. Singh, H. Sjödin, M. R. Smith, M. Sofiev, C. Sorensen, M. Springmann, J. D. Stowell, M. Tabatabaei, F. Tartarini, J. Taylor, C. Tonne, M. Treskova, J. A. Trinanes, A. Uppstu, N. Valdes-Ortega, F. Wagner, N. Watts, H. Whitcombe, R. Wood, P. Yang, Y. Zhang, S. Zhang, C. Zhang, S. Zhang, Q. Zhu, P. Gong, H. Montgomery, A. Costello, The 2025 report of the Lancet Countdown on health and climate change. Lancet 406, 2804–2857 (2025). 10.1016/S0140-6736(25)01919-1 41175887

[2] C. Mora, T. McKenzie, I. M. Gaw, J. M. Dean, H. von Hammerstein, T. A. Knudson, R. O. Setter, C. Z. Smith, K. M. Webster, J. A. Patz, E. C. Franklin, Over half of known human pathogenic diseases can be aggravated by climate change. Nat. Clim. Change 12, 869–875 (2022). 10.1038/s41558-022-01426-1 PMC9362357 35968032

[3] World Health Organization, World malaria report 2025: Addressing the threat of antimalarial drug resistance, (Geneva, 2025).

[4] S. Bhatt, P. W. Gething, O. J. Brady, J. P. Messina, A. W. Farlow, C. L. Moyes, J. M. Drake, J. S. Brownstein, A. G. Hoen, O. Sankoh, M. F. Myers, D. B. George, T. Jaenisch, G. R. Wint, C. P. Simmons, T. W. Scott, J. J. Farrar, S. I. Hay, The global distribution and burden of dengue. Nature 496, 504–507 (2013). 10.1038/nature12060 PMC3651993 23563266

[5] M. G. Guzman, M. Alvarez, S. B. Halstead, Secondary infection as a risk factor for dengue hemorrhagic fever/dengue shock syndrome: An historical perspective and role of antibody-dependent enhancement of infection. Arch. Virol. 158, 1445–1459 (2013). 10.1007/s00705-013-1645-3 23471635

[6] K. L. Anders, S. I. Hay, Lessons from malaria control to help meet the rising challenge of dengue. Lancet Infect. Dis. 12, 977–984 (2012). 10.1016/S1473-3099(12)70246-3 PMC3574272 23174383

[7] T. Alcayna, V. B. Rao, R. Lowe, Identifying the climate sensitivity of infectious diseases: A conceptual framework. Lancet Planet. Heath 9, 101291–101291 (2025). 10.1016/j.lanplh.2025.101291 40763757

[8] C. Putaporntip, S. Yanmanee, J. Somkuna, R. Rojrung, U. Pattanawong, S. Karapan, N. Kuamsab, C. W. Cheng, S. Jongwutiwes, Ecological complexity of zoonotic malaria in macaque natural hosts. Sci. Rep. 15, 4893 (2025). 10.1038/s41598-025-86415-y PMC11810990 39929916

[9] J. N. Mills, K. L. Gage, A. S. Khan, Potential influence of climate change on vector-borne and zoonotic diseases: A review and proposed research plan. Environ. Health Perspect. 118, 1507–1514 (2010). 10.1289/ehp.0901389 PMC2974686 20576580

[10] M. N. Bayoh, S. W. Lindsay, Temperature-related duration of aquatic stages of the Afrotropical malaria vector mosquito *Anopheles gambiae* in the laboratory. Med. Vet. Entomol. 18, 174–179 (2004). 10.1111/j.0269-283X.2004.00495.x 15189243

[11] W. Tun-Lin, T. R. Burkot, B. H. Kay, Effects of temperature and larval diet on development rates and survival of the dengue vector *Aedes aegypti* in north Queensland, Australia. Med. Vet. Entomol. 14, 31–37 (2000). 10.1046/j.1365-2915.2000.00207.x 10759309

[12] O. J. Brady, N. Golding, D. M. Pigott, M. U. G. Kraemer, J. P. Messina, R. C. Reiner Jr., T. W. Scott, D. L. Smith, P. W. Gething, S. I. Hay, Global temperature constraints on *Aedes aegypti* and *Ae. albopictus* persistence and competence for dengue virus transmission. Parasit. Vectors 7, 338 (2014). 10.1186/1756-3305-7-338 PMC4148136 25052008

[13] L. L. M. Shapiro, S. A. Whitehead, M. B. Thomas, Quantifying the effects of temperature on mosquito and parasite traits that determine the transmission potential of human malaria. PLoS Biol. 15, e2003489 (2017). 10.1371/journal.pbio.2003489 PMC5658182 29036170

[14] Y. L. Hii, R. A. Zaki, N. Aghamohammadi, J. Rocklöv, Research on climate and dengue in Malaysia: A systematic review. Curr. Environ. Health Rep. 3, 81–90 (2016). 10.1007/s40572-016-0078-z PMC4789198 26931438

[15] A. B. de Garín, R. A. Bejarán, A. E. Carbajo, S. C. de Casas, N. J. Schweigmann, Atmospheric control of *Aedes aegypti* populations in Buenos Aires (Argentina) and its variability. Int. J. Biometeorol. 44, 148–156 (2000). 10.1007/s004840000051 11049004

[16] T. W. Scott, P. H. Amerasinghe, A. C. Morrison, L. H. Lorenz, G. G. Clark, D. Strickman, P. Kittayapong, J. D. Edman, Longitudinal studies of *Aedes aegypti* (Diptera: Culicidae) in Thailand and Puerto Rico: Blood feeding frequency. J. Med. Entomol. 37, 89–101 (2000). 10.1603/0022-2585-37.1.89 15218911

[17] T. W. Scott, W. Takken, Feeding strategies of anthropophilic mosquitoes result in increased risk of pathogen transmission. Trends Parasitol. 28, 114–121 (2012). 10.1016/j.pt.2012.01.001 22300806

[18] M. Chan, M. A. Johansson, The incubation periods of dengue viruses. PLOS ONE 7, e50972 (2012). 10.1371/journal.pone.0050972 PMC3511440 23226436

[19] E. A. Mordecai, K. P. Paaijmans, L. R. Johnson, C. Balzer, T. Ben-Horin, E. de Moor, A. McNally, S. Pawar, S. J. Ryan, T. C. Smith, K. D. Lafferty, Optimal temperature for malaria transmission is dramatically lower than previously predicted. Ecol. Lett. 16, 22–30 (2013). 10.1111/ele.12015 23050931

[20] E. A. Mordecai, J. M. Cohen, M. V. Evans, P. Gudapati, L. R. Johnson, C. A. Lippi, K. Miazgowicz, C. C. Murdock, J. R. Rohr, S. J. Ryan, V. Savage, M. S. Shocket, A. Stewart Ibarra, M. B. Thomas, D. P. Weikel, Detecting the impact of temperature on transmission of Zika, dengue, and chikungunya using mechanistic models. PLoS Negl. Trop. Dis. 11, e0005568 (2017). 10.1371/journal.pntd.0005568 PMC5423694 28448507

[21] I. Trejo, M. Barnard, J. A. Spencer, J. Keithley, K. M. Martinez, I. Crooker, N. Hengartner, E. O. Romero-Severson, C. Manore, Changing temperature profiles and the risk of dengue outbreaks. PLOS Clim. 2, e0000115 (2023).

[22] T. A. Abeku, A. Iwegbu, S. Ogunmola, A. O. Oluwagbemiga, Seasonal climate patterns and their impact on mosquito net use in Nigeria: Evidence from repeated surveys. Malar. J. 25, 65 (2025). 10.1186/s12936-025-05761-4 PMC12853730 41455965

[23] L. Lambrechts, K. P. Paaijmans, T. Fansiri, L. B. Carrington, L. D. Kramer, M. B. Thomas, T. W. Scott, Impact of daily temperature fluctuations on dengue virus transmission by *Aedes aegypti*. Proc. Natl. Acad. Sci.U.S.A. 108, 7460–7465 (2011). 10.1073/pnas.1101377108 PMC3088608 21502510

[24] J. Liu-Helmersson, H. Stenlund, A. Wilder-Smith, J. Rocklöv, Vectorial capacity of *Aedes aegypti*: Effects of temperature and implications for global dengue epidemic potential. PLOS ONE 9, e89783 (2014). 10.1371/journal.pone.0089783 PMC3946027 24603439

[25] J. H. Huber, M. L. Childs, J. M. Caldwell, E. A. Mordecai, Seasonal temperature variation influences climate suitability for dengue, chikungunya, and Zika transmission. PLoS Negl. Trop. Dis. 12, e0006451 (2018). 10.1371/journal.pntd.0006451 PMC5963813 29746468

[26] K. P. Paaijmans, A. F. Read, M. B. Thomas, Understanding the link between malaria risk and climate. Proc. Natl. Acad. Sci.U.S.A. 106, 13844–13849 (2009). 10.1073/pnas.0903423106 PMC2720408 19666598

[27] P. W. Thorne, M. G. Donat, R. J. H. Dunn, C. N. Williams, L. V. Alexander, J. Caesar, I. Durre, I. Harris, Z. Hausfather, P. D. Jones, M. J. Menne, R. Rohde, R. S. Vose, R. Davy, A. M. G. Klein-Tank, J. H. Lawrimore, T. C. Peterson, J. J. Rennie, Reassessing changes in diurnal temperature range: Intercomparison and evaluation of existing global data set estimates. J. Geophys. Res. Atmos. 121, 5138–5158 (2016).

[28] N. L. Dennington, M. K. Grossman, J. L. Teeple, L. R. Johnson, M. S. Shocket, E. A. McGraw, M. B. Thomas, Phenotypic variation in populations of the mosquito vector, *Aedes aegypti*, and implications for predicting the effects of temperature and climate change on dengue transmission. PLoS Negl. Trop. Dis. 19, e0013623 (2025). 10.1371/journal.pntd.0013623 PMC12643283 41252453

[29] J. Shaman, J. F. Day, Reproductive phase locking of mosquito populations in response to rainfall frequency. PLOS ONE 2, e331 (2007). 10.1371/journal.pone.0000331 PMC1824708 17396162

[30] S. I. Hay, C. A. Guerra, A. J. Tatem, P. M. Atkinson, R. W. Snow, Urbanization, malaria transmission and disease burden in Africa. Nat. Rev. Microbiol. 3, 81–90 (2005). 10.1038/nrmicro1069 PMC3130901 15608702

[31] Q. Liu, Y. Wang, J. Deng, W. Yan, C. Qin, M. Du, M. Liu, J. Liu, Association of temperature and precipitation with malaria incidence in 57 countries and territories from 2000 to 2019: A worldwide observational study. J. Glob. Health 14, 04021 (2024). 10.7189/jogh.14.04021 PMC10882640 38385445

[32] F. Kane, M. Toure, N. Sogoba, B. Traore, M. Keita, D. Konate, S. I. Diawara, D. Sanogo, S. Keita, I. Sanogo, C. O. Doumbia, B. Keïta, A. S. Traoré, I. Sissoko, H. Coulibaly, S. M. B. Thiam, A. Barry, J. G. Shaffer, M. Diakite, S. Doumbia, Modeling clinical malaria episodes in different ecological settings in Mali, 2018-2022. IJID Reg. 10, 24–30 (2024). 10.1016/j.ijregi.2023.11.006 PMC10698665 38076024

[33] M. C. Thomson, S. J. Mason, T. Phindela, S. J. Connor, Use of rainfall and sea surface temperature monitoring for malaria early warning in Botswana. Am. J. Trop. Med. Hyg. 73, 214–221 (2005). 16014862

[34] M. C. Thomson, F. J. Doblas-Reyes, S. J. Mason, R. Hagedorn, S. J. Connor, T. Phindela, A. P. Morse, T. N. Palmer, Malaria early warnings based on seasonal climate forecasts from multi-model ensembles. Nature 439, 576–579 (2006). 10.1038/nature04503 16452977

[35] R. Gibb, F. J. Colón-González, P. T. Lan, P. T. Huong, V. S. Nam, V. T. Duoc, D. T. Hung, N. T. Dong, V. C. Chien, L. T. T. Trang, D. Kien Quoc, T. M. Hoa, N. H. Tai, T. T. Hang, G. Tsarouchi, E. Ainscoe, Q. Harpham, B. Hofmann, D. Lumbroso, O. J. Brady, R. Lowe, Interactions between climate change, urban infrastructure and mobility are driving dengue emergence in Vietnam. Nat. Commun. 14, 8179–8115 (2023). 10.1038/s41467-023-43954-0 PMC10713571 38081831

[36] R. Barrera, M. Amador, A. J. MacKay, Population dynamics of *Aedes aegypti* and dengue as influenced by weather and human behavior in San Juan, Puerto Rico. PLoS Negl. Trop. Dis. 5, e1378 (2011). 10.1371/journal.pntd.0001378 PMC3243685 22206021

[37] M. Pirani, C. Lorenz, T. S. de Azevedo, G. L. Barbosa, M. Blangiardo, F. Chiaravalloti-Neto, Effects of the El Niño-Southern Oscillation and seasonal weather conditions on *Aedes aegypti* infestation in the State of São Paulo (Brazil): A Bayesian spatio-temporal study. PLoS Negl. Trop. Dis. 18, e0012397 (2024). 10.1371/journal.pntd.0012397 PMC11392405 39264869

[38] K. P. Paaijmans, M. O. Wandago, A. K. Githeko, W. Takken, Unexpected high losses of *Anopheles gambiae* larvae due to rainfall. PLOS ONE 2, e1146 (2007). 10.1371/journal.pone.0001146 PMC2063461 17987125

[39] M. Kearney, W. P. Porter, C. Williams, S. Ritchie, A. A. Hoffmann, Integrating biophysical models and evolutionary theory to predict climatic impacts on species’ ranges: The dengue mosquito *Aedes aegypti* in Australia. Funct. Ecol. 23, 528–538 (2009).

[40] R. Lowe, S. A. Lee, K. M. O’Reilly, O. J. Brady, L. Bastos, G. Carrasco-Escobar, R. de Castro Catão, F. J. Colón-González, C. Barcellos, M. S. Carvalho, M. Blangiardo, H. Rue, A. Gasparrini, Combined effects of hydrometeorological hazards and urbanisation on dengue risk in Brazil: A spatiotemporal modelling study. Lancet Planet. Health 5, e209–e219 (2021). 10.1016/S2542-5196(20)30292-8 33838736

[41] R. Lowe, A. Gasparrini, C. J. Van Meerbeeck, C. A. Lippi, R. Mahon, A. R. Trotman, L. Rollock, A. Q. J. Hinds, S. J. Ryan, A. M. Stewart-Ibarra, Nonlinear and delayed impacts of climate on dengue risk in Barbados: A modelling study. PLoS Med. 15, e1002613 (2018). 10.1371/journal.pmed.1002613 PMC6049902 30016319

[42] O. R. Cawiding, S. Jeon, D. Tubera-Panes, A. A. de Los Reyes V, J. K. Kim, Disentangling climate’s dual role in dengue dynamics: A multiregion causal analysis study. Sci. Adv. 11, eadq1901 (2025). 10.1126/sciadv.adq1901 PMC11818013 39937893

[43] M. J. Bouma, C. Dye, Cycles of malaria associated with El Niño in Venezuela. JAMA 278, 1772–1774 (1997). 9388155

[44] E. O. Asare, A. M. Tompkins, A. Bomblies, A regional model for malaria vector developmental habitats evaluated using explicit pond-resolving surface hydrology simulations. PLoS One 11, e0150626 (2016). 10.1371/journal.pone.0150626 PMC4803214 27003834

[45] A. Bomblies, J.-B. Duchemin, E. A. B. Eltahir, A mechanistic approach for accurate simulation of village scale malaria transmission. Malar. J. 8, 223–223 (2009). 10.1186/1475-2875-8-223 PMC2761400 19799793

[46] A. Bomblies, Modeling the role of rainfall patterns in seasonal malaria transmission. Clim. Change 112, 673–685 (2012).

[47] J. J. Brown, M. Pascual, M. C. Wimberly, L. R. Johnson, C. C. Murdock, Humidity—The overlooked variable in the thermal biology of mosquito-borne disease. Ecol. Lett. 26, 1029–1049 (2023). 10.1111/ele.14228 PMC10299817 37349261

[48] S. Bhattacharya, C. Sharma, R. C. Dhiman, A. P. Mitra, Climate change and malaria in India. Curr. Sci. 90, 369–375 (2006).

[49] J. Babaie, M. Barati, M. Azizi, A. Ephtekhari, S. J. Sadat, A systematic evidence review of the effect of climate change on malaria in Iran. J. Parasit. Dis. 42, 331–340 (2018). 10.1007/s12639-018-1017-8 PMC6104236 30166779

[50] C. Duque, M. Lubinda, J. Matoba, C. Sing’anga, J. Stevenson, T. Shields, C. J. Shiff, Impact of aerial humidity on seasonal malaria: An ecological study in Zambia. Malar. J. 21, 325 (2022). 10.1186/s12936-022-04345-w PMC9652975 36369086

[51] E. Descloux, M. Mangeas, C. E. Menkes, M. Lengaigne, A. Leroy, T. Tehei, L. Guillaumot, M. Teurlai, A.-C. Gourinat, J. Benzler, A. Pfannstiel, J.-P. Grangeon, N. Degallier, X. De Lamballerie, Climate-based models for understanding and forecasting dengue epidemics. PLoS Negl. Trop. Dis. 6, e1470 (2012). 10.1371/journal.pntd.0001470 PMC3279338 22348154

[52] S. Hales, N. de Wet, J. Maindonald, A. Woodward, Potential effect of population and climate changes on global distribution of dengue fever: An empirical model. Lancet 360, 830–834 (2002). 10.1016/S0140-6736(02)09964-6 12243917

[53] C. A. Schmidt, G. Comeau, A. J. Monaghan, D. J. Williamson, K. C. Ernst, Effects of desiccation stress on adult female longevity in *Aedes aegypti* and *Ae. albopictus* (Diptera: Culicidae): Results of a systematic review and pooled survival analysis. Parasit. Vectors 11, 267–267 (2018). 10.1186/s13071-018-2808-6 PMC5918765 29695282

[54] D. F. Steinhoff, A. J. Monaghan, L. Eisen, M. J. Barlage, T. M. Hopson, I. Tarakidzwa, K. Ortiz-Rosario, S. Lozano-Fuentes, M. H. Hayden, P. E. Bieringer, C. M. Welsh Rodríguez, WHATCH’EM: A weather-driven energy balance model for determining water height and temperature in container habitats for *Aedes aegypti*. Earth Interact. 20, 1–31 (2016). 10.1175/EI-D-15-0048.1 PMC5672950 29123363

[55] S. A. Juliano, G. F. O’Meara, J. R. Morrill, M. M. Cutwa, Desiccation and thermal tolerance of eggs and the coexistence of competing mosquitoes. Oecologia 130, 458–469 (2002). 10.1007/s004420100811 PMC2944657 20871747

[56] P. Reiter, From Shakespeare to Defoe: Malaria in England in the Little Ice Age. Emerg. Infect. Dis. 6, 1–11 (2000). 10.3201/eid0601.000101 PMC2627969 10653562

[57] L. Hulden, L. Hulden, K. Helioevaara, Endemic malaria: An “indoor” disease in northern Europe. Historical data analysed. Malar. J. 4, 19–19 (2005). 10.1186/1475-2875-4-19 PMC1090613 15847704

[58] S. M. Omer, J. L. Cloudsley-Thompson, Dry season biology of *Anopheles gambiae* Giles in the Sudan. Nature 217, 879–880 (1968).

[59] B. Byttebier, M. S. D. Majo, S. Fischer, Hatching response of *Aedes aegypti* (Diptera: Culicidae) eggs at low temperatures: Effects of hatching media and storage conditions. J. Med. Entomol. 51, 97–103 (2014). 10.1603/me13066 24605458

[60] G. Obholz, G. San Blas, S. Fischer, A. Diaz, Winter survival of *Aedes aegypti* (Diptera: Culicidae) eggs at its southern limit distribution. Acta Trop. 231, 106471 (2022). 10.1016/j.actatropica.2022.106471 35430266

[61] L. P. Campbell, C. Luther, D. Moo-Llanes, J. M. Ramsey, R. Danis-Lozano, A. T. Peterson, Climate change influences on global distributions of dengue and chikungunya virus vectors. Philos. Trans. R Soc. Lond. B Biol. Sci. 370, 20140135 (2015). 10.1098/rstb.2014.0135 PMC4342968 25688023

[62] Y. Proestos, G. K. Christophides, K. Ergüler, M. Tanarhte, J. Waldock, J. Lelieveld, Present and future projections of habitat suitability of the Asian tiger mosquito, a vector of viral pathogens, from global climate simulation. Philos. Trans. R Soc. Lond. B Biol. Sci. 370, 20130554 (2015). 10.1098/rstb.2013.0554 PMC4342960 25688015

[63] K. M. Searle, D. E. Earland, A. Francisco Bibe, A. Novela, V. Muhiro, J. L. Ferrão, Long-lasting household damage from Cyclone Idai increases malaria risk in rural western Mozambique. Sci. Rep. 13, 21590–21510 (2023). 10.1038/s41598-023-49200-3 PMC10703775 38062239

[64] F. E. L. Otto, M. Zachariah, F. Saeed, A. Siddiqi, S. Kamil, H. Mushtaq, T. Arulalan, K. AchutaRao, S. T. Chaithra, C. Barnes, S. Philip, S. Kew, R. Vautard, G. Koren, I. Pinto, P. Wolski, M. Vahlberg, R. Singh, J. Arrighi, M. van Aalst, L. Thalheimer, E. Raju, S. Li, W. Yang, L. J. Harrington, B. Clarke, Climate change increased extreme monsoon rainfall, flooding highly vulnerable communities in Pakistan. Environ. Res. Clim. 2, 25001 (2023).

[65] World Health Organization, World malaria report 2023, (Geneva, 2023).

[66] M. J. Harris, J. T. Trok, K. S. Martel, M. J. Borbor-Cordova, N. S. Diffenbaugh, C. V. Munayco, A. G. Lescano, E. A. Mordecai, Extreme precipitation, exacerbated by anthropogenic climate change, drove Peru’s record-breaking 2023 dengue outbreak. One Earth 9, 101619 (2026).

[67] C. Li, Q. Zhao, Z. Zhao, Q. Liu, W. Ma, The association between tropical cyclones and dengue fever in the Pearl River Delta, China during 2013-2018: A time-stratified case-crossover study. PLoS Negl. Trop. Dis. 15, e0009776 (2021). 10.1371/journal.pntd.0009776 PMC8454958 34499666

[68] C. T. Codeco, S. S. Oliveira, D. A. C. Ferreira, T. I. S. Riback, L. S. Bastos, R. M. Lana, I. F. Almeida, V. B. Godinho, O. G. Cruz, F. C. Coelho, Fast expansion of dengue in Brazil. Lancet Reg. Health Am. 12, 100274–100274 (2022). 10.1016/j.lana.2022.100274 PMC9904033 36776428

[69] S. Banu, W. Hu, Y. Guo, S. Naish, S. Tong, Dynamic spatiotemporal trends of dengue transmission in the Asia-Pacific region, 1955-2004. PLOS ONE 9, e89440 (2014). 10.1371/journal.pone.0089440 PMC3933625 24586780

[70] C. Barcellos, V. Matos, R. M. Lana, R. Lowe, Climate change, thermal anomalies, and the recent progression of dengue in Brazil. Sci. Rep. 14, 5948–5949 (2024). 10.1038/s41598-024-56044-y PMC10928122 38467690

[71] M. Kretschmer, J. Collins, A. P. Dale, B. Garrett, L. Koski, K. Zabel, R. N. Staab, K. Turnbow, J. Nativio, K. Andrews, W. E. Smith, J. Townsend, N. Busser, J. Will, K. Burr, F. K. Jones, G. A. Santiago, K. A. Fitzpatrick, I. Ruberto, K. Fitzpatrick, J. R. White, L. Adams, R. H. Sunenshine, Notes from the Field: First evidence of locally acquired dengue virus infection—Maricopa County, Arizona, November 2022. MMWR Morb. Mortal. Wkly Rep. 72, 290–291 (2023). 10.15585/mmwr.mm7211a5 PMC10027408 36928186

[72] A. Cochet, C. Calba, F. Jourdain, G. Grard, G. A. Durand, A. Guinard, H. Noël, M. C. Paty, F. Franke, Autochthonous dengue in mainland France, 2022: Geographical extension and incidence increase. Euro Surveill. 27, 2200818 (2022). 10.2807/1560-7917.ES.2022.27.44.2200818 PMC9635021 36330819

[73] B. D. Pandey, A. Costello, The dengue epidemic and climate change in Nepal. Lancet 394, 2150–2151 (2019). 10.1016/S0140-6736(19)32689-3 31839187

[74] J. Mariën, E. Mukomena, V. M. Tevuzula, H. Leirs, T. Huyse, A century of medical records reveal earlier onset of the malaria season in Haut-Katanga induced by climate change. BMJ Glob. Health 9, e015375 (2024). 10.1136/bmjgh-2024-015375 PMC11499855 39438071

[75] M. E. Loevinsohn, Climatic warming and increased malaria incidence in Rwanda. Lancet 343, 714–718 (1994). 10.1016/s0140-6736(94)91586-5 7907685

[76] A. S. Siraj, M. Santos-Vega, M. J. Bouma, D. Yadeta, D. R. Carrascal, M. Pascual, Altitudinal changes in malaria incidence in highlands of Ethiopia and Colombia. Science 343, 1154–1158 (2014). 10.1126/science.1244325 24604201

[77] M. J. Bouma, M. Pascual, in *Climate Change and Global Health* (CABI Wallingford UK, 2014), pp. 65–76.

[78] G. Zhou, N. Minakawa, A. K. Githeko, G. Yan, H. R. Herren, Association between climate variability and malaria epidemics in the East African highlands. Proc. Natl. Acad. Sci. U.S.A. 101, 2375–2380 (2004). 10.1073/pnas.0308714100 PMC356958 14983017

[79] C. J. Carlson, E. Bannon, E. Mendenhall, T. Newfield, S. Bansal, Rapid range shifts in African *Anopheles* mosquitoes over the last century. Biol. Lett. 19, 20220365 (2023). 10.1098/rsbl.2022.0365 PMC9929507 36789530

[80] P. Reiter, Global warming and malaria: knowing the horse before hitching the cart. Malar. J. 7, S3 (2008). 10.1186/1475-2875-7-S1-S3 PMC2604878 19091037

[81] S. I. Hay, J. Cox, D. J. Rogers, S. E. Randolph, D. I. Stern, G. D. Shanks, M. F. Myers, R. W. Snow, Climate change and the resurgence of malaria in the East African highlands. Nature 415, 905–909 (2002). 10.1038/415905a PMC3164800 11859368

[82] D. I. Stern, P. W. Gething, C. W. Kabaria, W. H. Temperley, A. M. Noor, E. A. Okiro, G. D. Shanks, R. W. Snow, S. I. Hay, Temperature and malaria trends in highland East Africa. PLOS ONE 6, e24524 (2011). 10.1371/journal.pone.0024524 PMC3174181 21935416

[83] S. Hales, P. Weinstein, Y. Souares, A. Woodward, El Niño and the dynamics of vectorborne disease transmission. Environ. Health Perspect. 107, 99–102 (1999). 10.1289/ehp.9910799 PMC1566321 9924003

[84] A. M. Stewart-Ibarra, R. Lowe, Climate and non-climate drivers of dengue epidemics in southern coastal ecuador. Am. J. Trop. Med. Hyg. 88, 971–981 (2013). 10.4269/ajtmh.12-0478 PMC3752767 23478584

[85] J.-P. Chretien, A. Anyamba, J. Small, S. Britch, J. L. Sanchez, A. C. Halbach, C. Tucker, K. J. Linthicum, Global climate anomalies and potential infectious disease risks: 2014-2015. PLoS Curr. 7, (2015). 10.1371/currents.outbreaks.95fbc4a8fb4695e049baabfc2fc8289f. PMC4323421 25685635

[86] M. C. Thomson, Á. G. Muñoz, R. Cousin, J. Shumake-Guillemot, Climate drivers of vector-borne diseases in Africa and their relevance to control programmes. Infect. Dis. Poverty 7, 81–22 (2018). 10.1186/s40249-018-0460-1 PMC6085673 30092816

[87] Y. Chen, Y. Xu, L. Wang, Y. Liang, N. Li, J. Lourenço, Y. Yang, Q. Lin, L. Wang, H. Zhao, B. Cazelles, H. Song, Z. Liu, Z. Wang, O. J. Brady, S. Cauchemez, H. Tian, Indian Ocean temperature anomalies predict long-term global dengue trends. Science 384, 639–646 (2024). 10.1126/science.adj4427 38723095

[88] J. M. Caldwell, A. D. LaBeaud, E. F. Lambin, A. M. Stewart-Ibarra, B. A. Ndenga, F. M. Mutuku, A. R. Krystosik, E. B. Ayala, A. Anyamba, M. J. Borbor-Cordova, R. Damoah, E. N. Grossi-Soyster, F. H. Heras, H. N. Ngugi, S. J. Ryan, M. M. Shah, R. Sippy, E. A. Mordecai, Climate predicts geographic and temporal variation in mosquito-borne disease dynamics on two continents. Nat. Commun. 12, 1233 (2021). 10.1038/s41467-021-21496-7 PMC7902664 33623008

[89] S. F. McGough, L. Clemente, J. N. Kutz, M. Santillana, A dynamic, ensemble learning approach to forecast dengue fever epidemic years in Brazil using weather and population susceptibility cycles. J. R. Soc. Interface 18, 20201006 (2021). 10.1098/rsif.2020.1006 PMC8205538 34129785

[90] F. J. Colón-González, M. O. Sewe, A. M. Tompkins, H. Sjödin, A. Casallas, J. Rocklöv, C. Caminade, R. Lowe, Projecting the risk of mosquito-borne diseases in a warmer and more populated world: A multi-model, multi-scenario intercomparison modelling study. Lancet Planet. Heath 5, e404–e414 (2021). 10.1016/S2542-5196(21)00132-7 PMC8280459 34245711

[91] P. Martens, R. S. Kovats, S. Nijhof, P. de Vries, M. T. J. Livermore, D. J. Bradley, J. Cox, A. J. McMichael, Climate change and future populations at risk of malaria. Glob. Environ. Chang. 9, S89–S107 (1999).

[92] F. C. Tanser, B. Sharp, D. le Sueur, Potential effect of climate change on malaria transmission in Africa. Lancet 362, 1792–1798 (2003). 10.1016/S0140-6736(03)14898-2 14654317

[93] C. Caminade, S. Kovats, J. Rocklov, A. M. Tompkins, A. P. Morse, F. J. Colón-González, H. Stenlund, P. Martens, S. J. Lloyd, Impact of climate change on global malaria distribution. Proc. Natl. Acad. Sci. U.S.A. 111, 3286–3291 (2014). 10.1073/pnas.1302089111 PMC3948226 24596427

[94] V. Ermert, A. H. Fink, A. P. Morse, H. Paeth, The impact of regional climate change on malaria risk due to greenhouse forcing and land-use changes in tropical Africa. Environ. Health Perspect. 120, 77–84 (2012). 10.1289/ehp.1103681 PMC3261943 21900078

[95] C. C. Murdock, E. D. Sternberg, M. B. Thomas, Malaria transmission potential could be reduced with current and future climate change. Sci. Rep. 6, 27771 (2016). 10.1038/srep27771 PMC4914975 27324146

[96] S. J. Ryan, A. McNally, L. R. Johnson, E. A. Mordecai, T. Ben-Horin, K. Paaijmans, K. D. Lafferty, Mapping physiological suitability limits for malaria in africa under climate change. Vector Borne Zoonotic Dis. 15, 718–725 (2015). 10.1089/vbz.2015.1822 PMC4700390 26579951

[97] S. J. Ryan, C. A. Lippi, F. Zermoglio, Shifting transmission risk for malaria in Africa with climate change: A framework for planning and intervention. Malar. J. 19, 170–114 (2020). 10.1186/s12936-020-03224-6 PMC7193356 32357890

[98] H. M. Khormi, L. Kumar, Future malaria spatial pattern based on the potential global warming impact in South and Southeast Asia. Geospat. Health 11, 416 (2016). 10.4081/gh.2016.416 27903054

[99] H. Nissan, I. Ukawuba, M. Thomson, Climate-proofing a malaria eradication strategy. Malar. J. 20, 190–116 (2021). 10.1186/s12936-021-03718-x PMC8053291 33865383

[100] T. H. Jetten, D. A. Focks, Potential changes in the distribution of dengue transmission under climate warming. Am. J. Trop. Med. Hyg. 57, 285–297 (1997). 10.4269/ajtmh.1997.57.285 9311638

[101] J. Liu-Helmersson, Å. Brännström, M. O. Sewe, J. C. Semenza, J. Rocklöv, Estimating past, present, and future trends in the global distribution and abundance of the arbovirus vector *Aedes aegypti* under climate change scenarios. Front. Public Health 7, 148 (2019). 10.3389/fpubh.2019.00148 PMC6582658 31249824

[102] M. U. G. Kraemer, R. C. Reiner, O. J. Brady, J. P. Messina, M. Gilbert, D. M. Pigott, D. Yi, K. Johnson, L. Earl, L. B. Marczak, S. Shirude, N. Davis Weaver, D. Bisanzio, T. A. Perkins, S. Lai, X. Lu, P. Jones, G. E. Coelho, R. G. Carvalho, W. Van Bortel, C. Marsboom, G. Hendrickx, F. Schaffner, C. G. Moore, H. H. Nax, L. Bengtsson, E. Wetter, A. J. Tatem, J. S. Brownstein, D. L. Smith, L. Lambrechts, S. Cauchemez, C. Linard, N. R. Faria, O. G. Pybus, T. W. Scott, Q. Liu, H. Yu, G. R. W. Wint, S. I. Hay, N. Golding, Past and future spread of the arbovirus vectors *Aedes aegypti* and *Aedes albopictus*. Nat. Microbiol. 4, 854–863 (2019). 10.1038/s41564-019-0376-y PMC6522366 30833735

[103] S. J. Ryan, C. J. Carlson, E. A. Mordecai, L. R. Johnson, Global expansion and redistribution of *Aedes*-borne virus transmission risk with climate change. PLoS Negl. Trop. Dis. 13, e0007213 (2019). 10.1371/journal.pntd.0007213 PMC6438455 30921321

[104] J. P. Messina, O. J. Brady, N. Golding, M. U. G. Kraemer, G. R. W. Wint, S. E. Ray, D. M. Pigott, F. M. Shearer, K. Johnson, L. Earl, L. B. Marczak, S. Shirude, N. Davis Weaver, M. Gilbert, R. Velayudhan, P. Jones, T. Jaenisch, T. W. Scott, R. C. Reiner, S. I. Hay, The current and future global distribution and population at risk of dengue. Nat. Microbiol. 4, 1508–1515 (2019). 10.1038/s41564-019-0476-8 PMC6784886 31182801

[105] K. N. Kolivras, Changes in dengue risk potential in Hawaii, USA, due to climate variability and change. Climate Res. 42, 1–11 (2010).

[106] H. Liu, X. Huang, X. Guo, P. Cheng, H. Wang, L. Liu, C. Zang, C. Zhang, X. Wang, G. Zhou, M. Gong, Climate change and *Aedes albopictus* risks in China: Current impact and future projection. Infect. Dis. Poverty 12, 26–14 (2023). 10.1186/s40249-023-01083-2 PMC10037799 36964611

[107] H. Lee, J. E. Kim, S. Lee, C. H. Lee, Potential effects of climate change on dengue transmission dynamics in Korea. PLOS ONE 13, e0199205 (2018). 10.1371/journal.pone.0199205 PMC6023222 29953493

[108] L. E. Escobar, D. Romero-Alvarez, R. Leon, M. A. Lepe-Lopez, M. E. Craft, M. J. Borbor-Cordova, J.-C. Svenning, Declining prevalence of disease vectors under climate change. Sci. Rep. 6, 39150 (2016). 10.1038/srep39150 PMC5159793 27982119

[109] F. J. Colón-González, I. Harris, T. J. Osborn, C. S. S. Bernardo, C. A. Peres, P. R. Hunter, I. R. Lake, Limiting global-mean temperature increase to 1.5–2°C could reduce the incidence and spatial spread of dengue fever in Latin America. Proc. Natl. Acad. Sci. U.S.A. 115, 6243–6248 (2018). 10.1073/pnas.1718945115 PMC6004471 29844166

[110] L. Fischer, N. Gültekin, M. B. Kaelin, J. Fehr, P. Schlagenhauf, Rising temperature and its impact on receptivity to malaria transmission in Europe: A systematic review. Travel Med. Infect. Dis. 36, 101815 (2020). 10.1016/j.tmaid.2020.101815 32629138

[111] D. J. Rogers, S. E. Randolph, The global spread of malaria in a future, warmer world. Science 289, 1763–1766 (2000). 10.1126/science.289.5485.1763 10976072

[112] J. Liu-Helmersson, M. Quam, A. Wilder-Smith, H. Stenlund, K. Ebi, E. Massad, J. Rocklöv, Climate change and *Aedes* vectors: 21st century projections for dengue transmission in europe. EBioMedicine 7, 267–277 (2016). 10.1016/j.ebiom.2016.03.046 PMC4909611 27322480

[113] S. M. Thomas, D. Fischer, S. Fleischmann, T. Bittner, C. Beierkuhnlein, Risk assessment of dengue virus amplification in Europe based on spatio-temporal high resolution climate change projections. Erdkunde 65, 137–150 (2011).

[114] F. Schaffner, A. Mathis, Dengue and dengue vectors in the WHO European region: Past, present, and scenarios for the future. Lancet Infect. Dis. 14, 1271–1280 (2014). 10.1016/S1473-3099(14)70834-5 25172160

[115] Z. Farooq, L. Segelmark, J. Rocklöv, K. Lillepold, M. O. Sewe, O. J. T. Briet, J. C. Semenza, Impact of climate and Aedes albopictus establishment on dengue and Chikungunya outbreaks in Europe: A time-to-event analysis. Lancet Planet. Heath 9, e374–e383 (2025). 10.1016/S2542-5196(25)00059-2 40381632

[116] M. Bouzid, F. J. Colón-González, T. Lung, I. R. Lake, P. R. Hunter, Climate change and the emergence of vector-borne diseases in Europe: Case study of dengue fever. BMC Public Health 14, 781–781 (2014). 10.1186/1471-2458-14-781 PMC4143568 25149418

[117] J. A. Patz, D. Campbell-Lendrum, T. Holloway, J. A. Foley, Impact of regional climate change on human health. Nature 438, 310–317 (2005). 10.1038/nature04188 16292302

[118] P. E. Parham, J. Waldock, G. K. Christophides, D. Hemming, F. Agusto, K. J. Evans, N. Fefferman, H. Gaff, A. Gumel, S. LaDeau, S. Lenhart, R. E. Mickens, E. N. Naumova, R. S. Ostfeld, P. D. Ready, M. B. Thomas, J. Velasco-Hernandez, E. Michael, Climate, environmental and socio-economic change: Weighing up the balance in vector-borne disease transmission. Philos. Trans. R Soc. Lond. B Biol. Sci. 370, 20130551 (2015). 10.1098/rstb.2013.0551 PMC4342957 25688012

[119] J. Rocklöv, R. Dubrow, Climate change: An enduring challenge for vector-borne disease prevention and control. Nat. Immunol. 21, 479–483 (2020). 10.1038/s41590-020-0648-y PMC7223823 32313242

[120] T. K. Yamana, A. Bomblies, E. A. B. Eltahir, Climate change unlikely to increase malaria burden in West Africa. Nat. Clim. Change 6, 1009–1013 (2016).

[121] A. J. Tatem, S. I. Hay, D. J. Rogers, Global traffic and disease vector dispersal. Proc. Natl. Acad. Sci. U.S.A. 103, 6242–6247 (2006). 10.1073/pnas.0508391103 PMC1435368 16606847

[122] A. Wesolowski, T. Qureshi, M. F. Boni, P. R. Sundsøy, M. A. Johansson, S. B. Rasheed, K. Engø-Monsen, C. O. Buckee, Impact of human mobility on the emergence of dengue epidemics in Pakistan. Proc. Natl. Acad. Sci. U.S.A. 112, 11887–11892 (2015). 10.1073/pnas.1504964112 PMC4586847 26351662

[123] P. Thongsripong, J. M. Hyman, D. D. Kapan, S. N. Bennett, Human–mosquito contact: A missing link in our understanding of mosquito-borne disease transmission dynamics. Ann. Entomol. Soc. Am. 114, 397–414 (2021). 10.1093/aesa/saab011 PMC8266639 34249219

[124] A. Renard, F. Pérez Lombardini, M. Pacheco Zapata, T. Porphyre, A. Bento, G. Suzán, D. Roiz, B. Roche, A. Arnal, Interaction of human behavioral factors shapes the transmission of arboviruses by *Aedes* and *Culex* mosquitoes. Pathogens 12, 1421 (2023). 10.3390/pathogens12121421 PMC10746986 38133304

[125] Z. A. Bhutta, J. Sommerfeld, Z. S. Lassi, R. A. Salam, J. K. Das, Global burden, distribution, and interventions for infectious diseases of poverty. Infect. Dis. Poverty 3, 21 (2014). 10.1186/2049-9957-3-21 PMC4126350 25110585

[126] A. R. Magalhães, C. T. Codeço, J. C. Svenning, L. E. Escobar, P. Van de Vuurst, T. Gonçalves-Souza, Neglected tropical diseases risk correlates with poverty and early ecosystem destruction. Infect. Dis. Poverty 12, 32 (2023). 10.1186/s40249-023-01084-1 PMC10084676 37038199

[127] A. Béguin, S. Hales, J. Rocklöv, C. Åström, V. R. Louis, R. Sauerborn, The opposing effects of climate change and socio-economic development on the global distribution of malaria. Glob. Environ. Chang. 21, 1209–1214 (2011).

[128] D. Campbell-Lendrum, L. Manga, M. Bagayoko, J. Sommerfeld, Climate change and vector-borne diseases: What are the implications for public health research and policy? Philos. Trans. R Soc. Lond. B Biol. Sci. 370, 20130552 (2015). 10.1098/rstb.2013.0552 PMC4342958 25688013

[129] P. W. Gething, D. L. Smith, A. P. Patil, A. J. Tatem, R. W. Snow, S. I. Hay, Climate change and the global malaria recession. Nature 465, 342–345 (2010). 10.1038/nature09098 PMC2885436 20485434

[130] B. L. Sharp, I. Kleinschmidt, E. Streat, R. Maharaj, K. I. Barnes, D. N. Durrheim, F. C. Ridl, N. Morris, I. Seocharan, S. Kunene, J. J. P. L. Grange, J. D. Mthembu, F. Maartens, C. L. Martin, A. Barreto, Seven years of regional malaria control collaboration—Mozambique, South Africa, and Swaziland. Am. J. Trop. Med. Hyg. 76, 42–47 (2007). PMC3749812 17255227

[131] R. W. Snow, B. Sartorius, D. Kyalo, J. Maina, P. Amratia, C. W. Mundia, P. Bejon, A. M. Noor, The prevalence of *Plasmodium falciparum* in sub-Saharan Africa since 1900. Nature 550, 515–518 (2017). 10.1038/nature24059 PMC5660624 29019978

[132] D. J. Weiss, P. A. Dzianach, A. Saddler, J. Lubinda, A. Browne, M. McPhail, S. F. Rumisha, F. Sanna, Y. Gelaw, J. B. Kiss, S. Hafsia, R. Jayaseelen, H. S. Baggen, P. Amratia, A. Bertozzi-Villa, O. Nesbit, J. Whisnant, K. E. Battle, M. Nguyen, K. A. Alene, E. Cameron, M. A. Penny, S. Bhatt, D. L. Smith, T. L. Symons, J. F. Mosser, C. J. L. Murray, S. I. Hay, P. W. Gething, Mapping the global prevalence, incidence, and mortality of *Plasmodium falciparum* and *Plasmodium vivax* malaria, 2000-22: A spatial and temporal modelling study. Lancet 405, 979–990 (2025). 10.1016/S0140-6736(25)00038-8 PMC11928297 40056919

[133] World Health Organization, Global dengue surveillance. https://worldhealthorg.shinyapps.io/dengue_global/ [accessed 6 March 2026].

[134] Z. Zeng, J. Zhan, L. Chen, H. Chen, S. Cheng, Global, regional, and national dengue burden from 1990 to 2017: A systematic analysis based on the global burden of disease study 2017. EClinicalMedicine 32, 100712–100712 (2021). 10.1016/j.eclinm.2020.100712 PMC7910667 33681736

[135] J. Rocklöv, Y. Tozan, Climate change and the rising infectiousness of dengue. Emerg. Top. Life Sci. 3, 133–142 (2019). 10.1042/ETLS20180123 PMC7288996 33523146

[136] E. A. Mordecai, S. J. Ryan, J. M. Caldwell, M. M. Shah, A. D. LaBeaud, Climate change could shift disease burden from malaria to arboviruses in Africa. Lancet Planet. Heath 4, e416–e423 (2020). 10.1016/S2542-5196(20)30178-9 PMC7490804 32918887

[137] J. P. Daily, S. Parikh, Malaria. N. Engl. J. Med. 392, 1320–1333 (2025). 10.1056/NEJMra2405313 PMC12331251 40174226

[138] R. G. A. Feachem, I. Chen, O. Akbari, A. Bertozzi-Villa, S. Bhatt, F. Binka, M. F. Boni, C. Buckee, J. Dieleman, A. Dondorp, A. Eapen, N. Sekhri Feachem, S. Filler, P. Gething, R. Gosling, A. Haakenstad, K. Harvard, A. Hatefi, D. Jamison, K. E. Jones, C. Karema, R. N. Kamwi, A. Lal, E. Larson, M. Lees, N. F. Lobo, A. E. Micah, B. Moonen, G. Newby, X. Ning, M. Pate, M. Quiñones, M. Roh, B. Rolfe, D. Shanks, B. Singh, K. Staley, J. Tulloch, J. Wegbreit, H. J. Woo, W. Mpanju-Shumbusho, Malaria eradication within a generation: ambitious, achievable, and necessary. Lancet 394, 1056–1112 (2019). 10.1016/S0140-6736(19)31139-0 31511196

[139] O. Horstick, Y. Tozan, A. Wilder-Smith, Reviewing dengue: Still a neglected tropical disease? PLoS Negl. Trop. Dis. 9, e0003632 (2015). 10.1371/journal.pntd.0003632 PMC4415787 25928673

[140] D. J. Gubler, Dengue, urbanization and globalization: The unholy trinity of the 21st century. Trop. Med. Health 39, 3–11 (2011). 10.2149/tmh.2011-S05 PMC3317603 22500131

[141] Y. Li, F. Kamara, G. Zhou, S. Puthiyakunnon, C. Li, Y. Liu, Y. Zhou, L. Yao, G. Yan, X.-G. Chen, Urbanization increases *Aedes albopictus* larval habitats and accelerates mosquito development and survivorship. PLoS Negl. Trop. Dis. 8, e3301 (2014). 10.1371/journal.pntd.0003301 PMC4230920 25393814

[142] S. A. Fernandez, H. Sun, B. L. Dickens, L. C. Ng, A. R. Cook, J. T. Lim, Features of the urban environment associated with *Aedes aegypti* abundance in high-rise public apartments in Singapore: An environmental case-control study. PLoS Negl. Trop. Dis. 17, e0011075 (2023). 10.1371/journal.pntd.0011075 PMC9928025 36730440

[143] P.-C. Wu, J.-G. Lay, H.-R. Guo, C.-Y. Lin, S.-C. Lung, H.-J. Su, Higher temperature and urbanization affect the spatial patterns of dengue fever transmission in subtropical Taiwan. Sci. Total Environ. 407, 2224–2233 (2009). 10.1016/j.scitotenv.2008.11.034 19157509

[144] A. J. Tatem, P. W. Gething, D. L. Smith, S. I. Hay, Urbanization and the global malaria recession. Malar. J. 12, 133–133 (2013). 10.1186/1475-2875-12-133 PMC3639825 23594701

[145] V. Robert, K. Macintyre, J. Keating, J. F. Trape, J. B. Duchemin, M. Warren, J. C. Beier, Malaria transmission in urban sub-Saharan Africa. Am. J. Trop. Med. Hyg. 68, 169–176 (2003). 12641407

[146] Q. Qi, C. A. Guerra, C. L. Moyes, I. A. R. F. Elyazar, P. W. Gething, S. I. Hay, A. J. Tatem, The effects of urbanization on global *Plasmodium vivax* malaria transmission. Malar. J. 11, 403–403 (2012). 10.1186/1475-2875-11-403 PMC3528462 23217010

[147] T. Emiru, D. Getachew, M. Murphy, L. Sedda, L. A. Ejigu, M. G. Bulto, I. Byrne, M. Demisse, M. Abdo, W. Chali, A. Elliott, E. N. Vickers, A. Aranda-Díaz, L. Alemayehu, S. W. Behaksera, G. Jebessa, H. Dinka, T. Tsegaye, H. Teka, S. Chibsa, P. Mumba, S. Girma, J. Hwang, M. Yoshimizu, A. Sutcliffe, H. S. Taffese, G. A. Bayissa, S. Zohdy, J. E. Tongren, C. Drakeley, B. Greenhouse, T. Bousema, F. G. Tadesse, Evidence for a role of *Anopheles stephensi* in the spread of drug- and diagnosis-resistant malaria in Africa. Nat. Med. 29, 3203–3211 (2023). 10.1038/s41591-023-02641-9 PMC10719088 37884028

[148] S. N. Surendran, K. Sivabalakrishnan, A. Sivasingham, T. T. P. Jayadas, K. Karvannan, S. Santhirasegaram, K. Gajapathy, M. Senthilnanthanan, S. P. Karunaratne, R. Ramasamy, Anthropogenic factors driving recent range expansion of the malaria vector *Anopheles stephensi*. Front. Public Health 7, 53 (2019). 10.3389/fpubh.2019.00053 PMC6426791 30923705

[149] J. M. Cohen, D. L. Smith, C. Cotter, A. Ward, G. Yamey, O. J. Sabot, B. Moonen, Malaria resurgence: A systematic review and assessment of its causes. Malar. J. 11, 122–122 (2012). 10.1186/1475-2875-11-122 PMC3458906 22531245

[150] T. L. Symons, A. Moran, A. Balzarolo, C. Vargas, M. Robertson, J. Lubinda, A. Saddler, M. McPhail, J. Harris, J. Rozier, A. Browne, P. Amratia, A. Bertozzi-Villa, S. Bhatt, E. Cameron, N. Golding, D. L. Smith, A. M. Noor, S. F. Rumisha, M. D. Palmer, D. J. Weiss, N. Desai, D. Potere, N. Sukitsch, W. Woods, P. W. Gething, Projected impacts of climate change on malaria in Africa. Nature 651, 390–396 (2026). 10.1038/s41586-025-10015-z PMC12979204 41606340

[151] G. Paz-Bailey, L. E. Adams, J. Deen, K. B. Anderson, L. C. Katzelnick, Dengue. Lancet 403, 667–682 (2024). 10.1016/S0140-6736(23)02576-X PMC12372472 38280388

[152] E. G. Kallás, J. A. Moreira, E. G. Patiño, P. E. Braga, J. C. V. Tenório, L. B. de Oliveira Alves, V. Infante, D. H. R. Silveira, J. L. Miraglia, M. A. T. Cintra, D. B. Pereira, A. J. da Fonseca, R. Q. Gurgel, I. C.-B. Coelho, C. J. F. Fontes, E. T. A. Marques, G. A. S. Romero, M. M. Teixeira, A. M. Siqueira, V. S. Boaventura, F. Ramos, E. E. Júnior, J. C. de Moraes, C. F. Estofolete, A. C. Freitas, C. L. S. D. Santos, M. D. C. S. T. Timenetsky, S. S. Whitehead, A. P. Durbin, A. Esteves-Jaramillo, T. Shekar, J.-J. Lee, Q. Chen, J. Macey, S. Vaidya, B.-A. G. Coller, F. C. Boulos, M. L. Nogueira, M. V. G. de Lacerda, Long-term efficacy and safety of the single-dose tetravalent Butantan dengue vaccine. Nat. Med. 32, 1393–1400 (2026). 10.1038/s41591-026-04255-3 41781546

[153] T. Walker, P. H. Johnson, L. A. Moreira, I. Iturbe-Ormaetxe, F. D. Frentiu, C. J. McMeniman, Y. S. Leong, Y. Dong, J. Axford, P. Kriesner, A. L. Lloyd, S. A. Ritchie, S. L. O’Neill, A. A. Hoffmann, The wMel *Wolbachia* strain blocks dengue and invades caged *Aedes aegypti* populations. Nature 476, 450–453 (2011). 10.1038/nature10355 21866159

[154] J. Hemingway, The way forward for vector control. Science 358, 998–999 (2017). 10.1126/science.aaj1644 29170222

